# Harnessing Jasmonate Pathways: PgJAR1’s Impact on Ginsenoside Accumulation in Ginseng

**DOI:** 10.3390/plants14060847

**Published:** 2025-03-08

**Authors:** Ru Zhang, Chao Li, Rui Guo, Zhaoying Li, Bianling Zhang

**Affiliations:** 1 College of Materials and Chemical Engineering, Hunan Institute of Engineering, Xiangtan 411104, China; lichao200028@163.com (C.L.); eilanck001@outlook.com (R.G.); 15827204305@163.com (Z.L.); blzhang369@163.com (B.Z.); 2 Innovation Institute of Advanced Functional Materials, Hunan Institute of Engineering, Xiangtan 411104, China

**Keywords:** *PgJAR1*, GH3 family, JA, JA-Ile, *Panax ginseng*, ginsenosides

## Abstract

Ginsenosides, the most active components in *Panax ginseng*, exhibit pharmacological and therapeutic properties but are limited by their low abundance. Jasmonates (JAs), a class of stress-induced phytohormones, are integral in modulating plant defense responses and the biosynthesis of secondary metabolites, including ginsenosides. Jasmonoyl-isoleucine (JA-Ile), the primary bioactive JA compound, is biosynthesized by JA-Ile synthase 1 (JAR1). In this study, we cloned the 1555 bp *PgJAR1* gene from ginseng roots and analyzed its structure, enzyme activity, and expression pattern. The PgJAR1 protein encompasses all the hallmark elements characteristic of the GH3 family. It exhibits N/C-terminal domains analogous to ANL, three ATP/AMP-binding motifs, and distinct secondary structures: an N-terminal beta-barrel with beta-sheets and alpha-helices, and a C-terminal beta-sheet surrounded by alpha-helices, similarly to AtGH3.11/AtJAR1. The recombinant PgJAR1 enzyme expressed in *Escherichia coli* BL21 specifically catalyzed jasmonic acid (JA) to JA-Ile. *PgJAR1* is predominantly expressed in leaves and is upregulated by MeJA treatment. Moderate transient overexpression of *PgJAR1* promoted the biosynthesis of both JA-Ile and ginsenosides, highlighting the crucial role of *PgJAR1* in JA-Ile biosynthesis and its positive impact on ginsenoside accumulation. Nevertheless, elevated JA-Ile levels can impede cellular growth, reducing ginsenoside production. Consequently, balancing JA-Ile biosynthesis through *PgJAR1* expression is essential for optimizing ginseng cultivation and enhancing its medicinal properties. Modulating endogenous JA-Ile levels offers a strategy for increasing ginsenoside production in ginseng plants.

## 1. Introduction

*Panax ginseng*, a highly esteemed herbal medicine in oriental countries for thousands of years [1,2,3], contains significant clinical compounds such as ginsenosides, polysaccharides, peptides, and phenolic compounds [4,5,6,7]. Ginsenosides are the primary bioactive compounds responsible for eliciting its pharmacological effects [8,9,10,11]. Nevertheless, the extended growth cycle of ginseng, ranging from 4 to 6 years, along with the strict demands for suitable soil, climate, and environmental conditions in addition to the defensive nature of ginsenosides as secondary metabolites aimed at mitigating stress, results in a notably low content of these compounds in ginseng [12,13]. This significantly constrains the advancement and utilization of numerous crucial medications containing high-activity ginsenosides as their primary active components [14,15]. Consequently, it is imperative to investigate more efficient methodologies for the cultivation of ginseng cells or tissues to achieve optimal yields. However, the accumulation of ginsenosides is not exclusively dictated by cultivation practices; it is also significantly influenced by prolonged interactions with the environment. Environmental stressors and nutrient and hormone availability during the growth phase of ginseng play a crucial role in modulating ginsenoside biosynthesis.

The family of lipid-derived compounds known as jasmonates (JAs) is essential for regulating various processes in plant physiology, including growth, development, and response to environmental stresses [16]. Among the discovered JAs, jasmonyl-isoleucine (JA-Ile) is the most active key signaling molecule that stimulates the biosynthesis of defensive secondary metabolites in plants [17]. It is mainly synthesized outside the plant nucleus and then transported to the nucleus, activating the core transcription factor MYC of the JA signal transduction pathway. MYC then binds to various promoters of secondary metabolite synthase genes, activating their expression and regulating the biosynthesis of plant secondary metabolites [17,18,19]. The unique receptor system of JA was revealed to involve interactions between the F-box protein coronatine-insensitive 1 (COI1) and the repressor protein jasmonate-ZIM-domain (JAZ), resulting in the formation of the COI1-JAZ complex. The degradation of the JAZ repressor through ubiquitination by the 26S proteasome results in the activation of downstream gene expression that is typically inhibited by the JAZ repressor [20,21]. In rice, JA-Ile plays a pivotal role in defense responses, particularly in the production of phytoalexins, like sakuranetin, which are essential for combating stress-induced conditions and pathogen attacks. The enzyme OsJAR1 is primarily responsible for the biosynthesis of JA-Ile in rice, contributing significantly to the plant’s defense mechanisms [22,23]. Moreover, JA-Ile is involved in the regulation of macronutrient deficiency tolerance in rice by modulating the root system architecture, which enhances nutrient acquisition and use efficiency [24]. Additionally, JA-Ile is required for the production of flavonoid phytoalexins in rice, which are crucial in the defense against pathogens and stress conditions [25]. A semi-quantitative analysis utilizing electron microscopic autoradiography on atjar1-mutant *Arabidopsis* cells, which exhibit significant impairment in the biosynthesis of JA-Ile, demonstrates that JA-Ile is crucial for the efficient and rapid activation of the core JA signaling pathway [26,27]. The cytochrome P450 enzyme CYP94B3 in *Arabidopsis* is responsible for the oxidative catabolism of JA-Ile, converting it into 12-hydroxy-JA-Ile, which is involved in modulating JA signaling and wound responses [28]. In tomato, JA-Ile is integral to the regulation of defense responses against herbivores and pathogens. The cytochrome P450 monooxygenases SlCYP94B18 and SlCYP94B19 are involved in the catabolic turnover of JA-Ile in tomato leaves, which is crucial for maintaining the balance of JA signaling and ensuring effective defense responses [29]. Furthermore, JA-Ile is implicated in the systemic defense signaling in response to herbivory in *Nicotiana attenuata*, a close relative of the tomato, highlighting its role in long-distance signaling and defense coordination [30]. In the context of symbiotic interactions, JA-Ile signaling has been shown to influence the establishment and functioning of arbuscular mycorrhizal symbiosis and root nodule symbiosis. The modulation of these interactions by JA-Ile involves complex signaling pathways that integrate environmental cues and developmental signals to optimize plant growth and stress resilience [31].

Jasmonoyl-isoleucine synthase 1 (JAR1) encodes an ATP-dependent JA-amido synthetase, which plays a crucial role in the JA signaling pathway. This enzyme is responsible for the conjugation of JA to isoleucine, forming JA-Ile, a bioactive form of JA that regulates various plant responses to stress and development [32,33,34]. The importance of JAR1 in plant hormone signaling is underscored by its involvement in the modulation of JA levels, which are critical for plant defense mechanisms against herbivores and pathogens [35,36]. Moreover, the regulation of *JAR1* expression and activity is critical for the proper functioning of the JA signaling cascade. This highlights the enzyme’s significance not only in the biosynthesis of JAs but also in the broader context of plant signaling and adaptive mechanisms [37].

However, to date, the investigation of *JAR1* genes in plants has been limited to a small number of instances. Recent studies have highlighted the structural and functional characteristics of JAR1, revealing how it interacts with its substrates. The crystal structure of AtJAR1 has been elucidated, providing insights into the enzyme’s substrate specificity and catalytic mechanism. This structural information is vital for understanding how JAR1 contributes to the dynamic regulation of JA signaling in plants [38]. Additionally, the enzyme’s activity is influenced by various factors, including the presence of amino acids and other signaling molecules, which can modulate its function and, consequently, the levels of active JAs in the plant [39]. Moreover, the interplay between JAR1 and other enzymes in the JA biosynthetic and catabolic pathways illustrates a complex regulatory network that governs plant responses to environmental stimuli. For instance, the amidohydrolases IAR3 and ILL6 have been shown to participate in the turnover of JA-Ile, further emphasizing the intricate balance of hormone levels maintained by these enzymes [40,41]. Understanding the role of JAR1 and its interactions within this network is essential for advancing our knowledge of plant hormone signaling and its implications for plant growth and defense strategies.

The roles of endogenous JAs in the biosynthesis of secondary metabolites in plants has garnered considerable interest, particularly in ginseng research. Empirical evidence indicates that JA not only facilitates the synthesis of ginsenosides but is also intricately linked to plant growth and defense mechanisms. Initial investigations have identified the *PgLOX6* gene as pivotal in the biosynthesis of JAs. The lipoxygenase enzyme encoded by this gene enhances JA synthesis in ginseng, thereby augmenting its ginsenoside content. Transgenic overexpression of *PgLOX6* in *Arabidopsis* has been shown to significantly elevate levels of JA and MeJA. Furthermore, this overexpression correlates with the increased expression of genes associated with triterpene biosynthesis, underscoring the critical role of *PgLOX6* in JA synthesis and ginsenoside production [42]. In addition, JAs interact with endogenous hormone signaling pathways, thereby influencing the synthesis of secondary metabolites. Integrated transcriptomic and metabolomic analyses have revealed that ginsenoside accumulation can be modulated by using genes of the plant hormone signaling pathway. This suggests that JAs play a regulatory role in endogenous hormone signal transduction, consequently affecting the synthesis of secondary metabolites in ginseng [43].

In this study, the *JAR1* gene was successfully cloned and identified from *Panax* species for the first time, which was subsequently designated as *PgJAR1*. *PgJAR1* plays a pivotal role in the regulation of ginsenoside biosynthesis by activating the JA signaling pathway. Moderate transient overexpression of *PgJAR1* in *P. ginseng* leaves was observed to significantly enhance the biosynthesis and accumulation of ginsenosides. *PgJAR1* facilitates the conjugation of JA with Ile to form JA-Ile, thereby activating the JA signaling pathway and regulating the expression of downstream genes associated with saponin synthesis. This discovery not only elucidates the essential function of *PgJAR1* in ginsenoside biosynthesis but also provides novel insights into optimizing ginsenoside production. By manipulating the expression or activity of *PgJAR1* to modulate JA-Ile levels, it may be possible to develop more efficient cultivation methods and to breed ginseng varieties with elevated levels of bioactive ginsenosides.

## 2. Results

### 2.1. Chromosomal Distribution Analysis of the GH3 Family in P. ginseng

**Figure 1:**
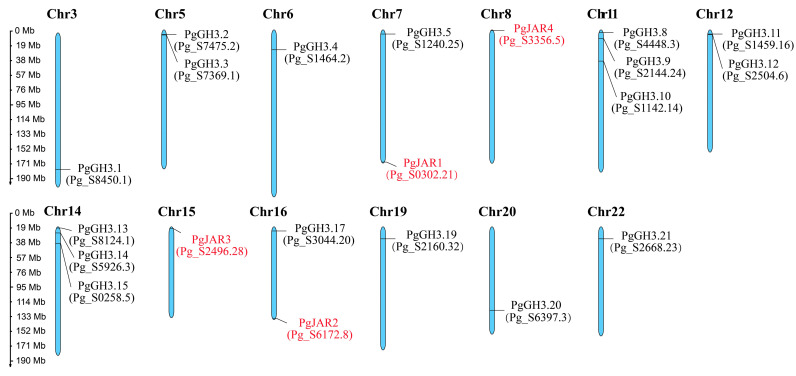
Chromosomal distribution of 21 *GH3* genes in *P. ginseng*. Chromosome numbers are listed above each chromosome, and chromosome lengths in megabases (Mb) are shown on the left side of the figure. The *PgJAR* genes are highlighted in red. “Chr” denotes chromosome.

In order to identify genes associated with JA-Ile synthesis, ginseng hairy roots were treated with methyl jasmonate (MeJA). Transcriptome analysis revealed 21 *GH3* (Gretchen Hagen 3) family genes, of which 4 were identified as *JAR* genes; these were designated as *PgJAR1*, *PgJAR2*, *PgJAR3*, and *PgJAR4* based on their sequence homology and phylogenetic relationships with known *JAR* genes in other plant species. The sequences of these genes are provided in Supplementary Table S1. Our analysis demonstrated a heterogeneous distribution of the *GH3* gene family across the 24 chromosomes of *P*. *ginseng*, as illustrated in Figure 1. *GH3* genes were identified on only 13 chromosomes, with the four *PgJAR* genes specifically localized to chromosomes 7, 8, 15, and 16. Notably, chromosomes 11 and 14 exhibited the highest gene density, followed by chromosomes 5 and 12, whereas the remaining chromosomes harbored only a single gene. Intriguingly, no *GH3* gene family members were detected on the other 11 chromosomes.

### 2.2. Cloning of the PgJAR1 Gene from P. ginseng and Its Sequence Analysis

**Figure 2:**
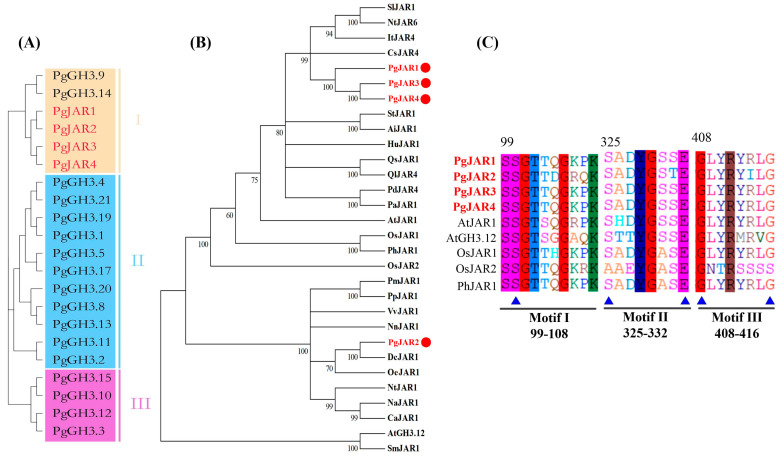
Sequence analysis of PgJAR1. (**A**) Phylogenetic tree constructed using the GH3 proteins of *P. ginseng* (PgGH3). (**B**) Phylogenetic tree constructed using PgJARs and their orthologous proteins in several other species. The four PgJARs are marked as a red solid circle. The orthologous proteins are from other 26 plant species, including *A. thaliana* and *O. sativa* (for a full listing, see Supplementary Tables S1 and S2). (**C**) Conserved domains of PgJARs compared with orthologous proteins from other species. *Arabidopsis thaliana* (At), *Oryza sativa* (Os), *Panicum hallii* (Ph). Residues marked with a blue triangle below are the ATP-binding residues in the JAR proteins.

To investigate the diversity of *GH3* genes in *P. ginseng*, a group classification analysis was performed. The results demonstrated that all *GH3* genes were categorized into three distinct groups (I, II, and III), with all the PgJARs specifically clustered into group I (Figure 2A). To further evaluate the evolutionary relationships of the PgJARs, a phylogenetic tree was constructed using the amino acid sequences of the 4 *PgJAR* genes (*PgJAR1*, *PgJAR2*, *PgJAR3*, and *PgJAR4*) from ginseng alongside 26 *JAR* genes from other species. Detailed sequence information and accession numbers are available in Supplementary Tables S1 and S2. The analysis revealed that PgJAR1 shares 53.5% to 84.6% amino acid sequence homology with other JAR proteins (Figure 2B). Phylogenetic analysis indicated that PgJAR1 is most closely related to PgJAR3 and PgJAR4, but the most similar sequences in a different species are CsJAR4 (*Camellia sinensis*) and SlJAR1 (*Solanum lycopersicum*), both of which belong to the GH3 I subfamily. This close phylogenetic relationship suggests functional conservation among these JAR proteins, particularly in their roles in relation to JA signaling and metabolism. The findings provide insights into the evolutionary diversification of *JAR* genes and highlight the potential functional similarities between PgJAR1 and its orthologs in other plant species, which may further elucidate their roles in regulating JA-mediated physiological processes.

The cDNA contains a 1755 bp open reading frame (ORF), which encodes a 584-amino acid peptide with a molecular weight of 65.72 kDa and an isoelectric point of 5.49. The amino acid sequences of the four PgJARs exhibit all the characteristic elements of the GH3 enzyme family, featuring both an N-terminal and a C-terminal domain typical of the adenylating firefly luciferase, which belongs to the ANL enzyme superfamily. In the case of PgJAR1, three short conserved motifs involved in ATP/AMP binding were observed: motif I (SSGTTQGKPK, 99–108), motif II (SADYGSSE, 325–332), and motif III (GLYRYRLGD, 408–416) (Figure 2C). Compared to PgJAR1, the sequences of motifs I, II and III in PgJAR2, PgJAR3, and PgJAR4 were identical, except for PgJAR2, which had D104, R106, and Q107 in motif I, T331 in motif II, and I413 in motif III. While OsJAR2 possesses two unique ATP-binding residues (A333 and S414) that differ from other JARs, the remaining ATP-binding residues are highly conserved across the family. Motif I represents a canonical phosphate-binding (P) loop, where the hydroxyl groups of S99 and S100 interact with the α-phosphate. Motif II adopts a β-turn conformation, with S325 forming a hydrogen bond with the α-phosphate group and E332 engaging in stacking interactions with the adenine ring. Motif III features a conserved aspartate residue (D332) that interacts with the nucleotide ribose. Structurally, the N-terminal domain of PgJAR1 is characterized by a beta-barrel structure and two beta-sheets, which are flanked by alpha-helices. The C-terminal domain consists of a single four-stranded beta-sheet bracketed by two alpha-helices on each side.

**Figure 3:**
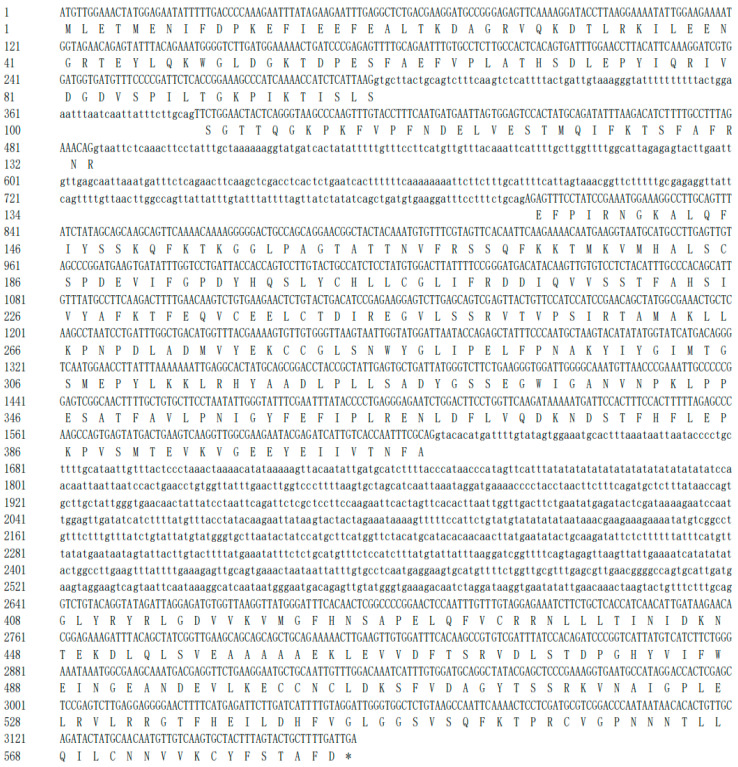
The genomic sequence structure of *PgJAR1* (capital letters represent exons; lowercase letters indicate introns). * represents a stop codon.

Among the four identified *PgJAR* genes, the *PgJAR1* gene is significantly induced by MeJA, while the expression of the other three *PgJAR* genes is suppressed by MeJA (Supplementary Figure S1). This suggests that *PgJAR1* may be involved in the JA signaling pathway, making it a candidate gene for further investigation. The full-length coding sequence (CDS) of *P. ginseng JAR1* (*PgJAR1*) was successfully cloned based on the sequence information from transcriptomic data of MeJA-induced ginseng hairy roots. Sequence alignment analysis revealed significant differences in DNA sequence identity among the four *PgJAR* homologs. *PgJAR3* and *PgJAR4* exhibited a high sequence identity of 98.9%, suggesting that they may have originated from a recent gene duplication event and could share similar functional roles. In contrast, *PgJAR3* and *PgJAR4* showed lower sequence identities with *PgJAR1*, at 89.1% and 89.38%, respectively. Furthermore, *PgJAR1* and *PgJAR2* displayed the lowest sequence identity at 65.1%, indicating potential functional divergence between these two genes. The high sequence conservation between *PgJAR3* and *PgJAR4* implies that they may have redundant or overlapping functions, while the lower identity between *PgJAR1* and *PgJAR2* suggests distinct roles in the JA signaling pathway. The complete genomic DNA of *PgJAR1* was successfully cloned using genomic DNA as the template, and the bioinformatics analysis indicates that the sequence contains three introns and four exons (Figure 3).

**Figure 4:**
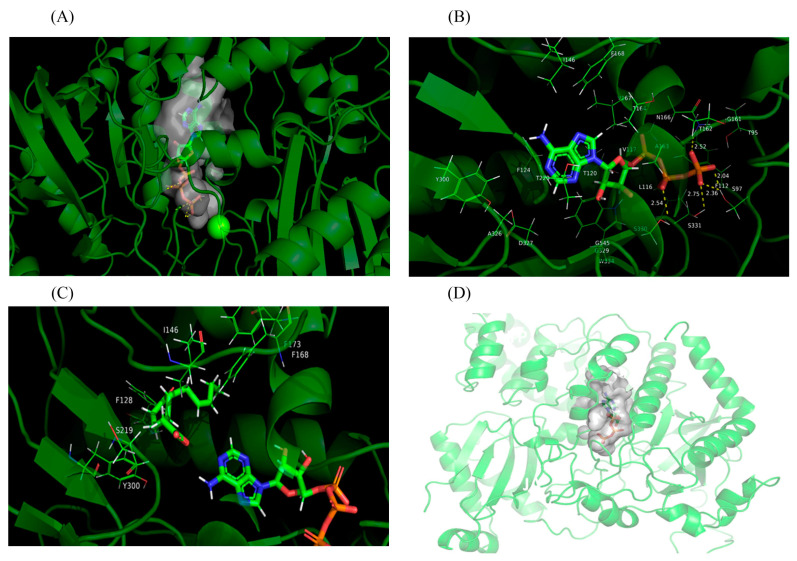
Three-dimensional (3D) structure of PgJAR1 with JA and ATP. (**A**) The binding pocket of ATP in the PgJAR1 protein. (**B**) Molecular docking analysis of PgJAR1 and ATP. (**C**) Close-up view of the JA- and ATP-binding sites of PgJAR1. (**D**) Schematic diagram of the escape door for diphosphate.

Based on the crystal structure of far-red-insensitive 219 (FIN219)/JAR1 (5ECI), the 3D structure model of PgJAR1 (Figure 4A) was constructed to identify the key residues involved in its catalytic activities. The predicted secondary structure of PgJAR1, as determined by the Swiss-Model program, showed 66.43% identity with that of FIN219. The secondary and tertiary structures were accurately predicted. Molecular docking analysis of the 3D structure model (PDB file) of PgJAR1 was conducted using JA and ATP as substrates with AutoDock. The docking results were evaluated based on the minimum energy value. The docking analysis of ATP revealed a maximum binding affinity of 8.76 kCal/mol between ATP and PgJAR1. Notably, the phosphate group exhibited extensive hydrogen-bonding interactions with several amino acid residues, including T162 (2.52 Å), S97 (2.36 Å, 2.04 Å), S331 (2.75 Å), and S330 (2.54 Å). The PgJAR1 protein possesses a relatively larger internal cavity, which becomes more accommodating for JA and Ile following the hydrolysis of ATP to AMP, facilitating the linkage of Ile to the carboxyl group of JA, as illustrated in Figure 4B. There is an observable exit at the site where the phosphate group binds within the protein, which facilitates the dissociation of diphosphate from ATP and its subsequent release through the adjacent exit (Figure 4C). The maximum binding energy for JA docking into the internal active pocket of the PgJAR1 is calculated to be 7.98 kCal/mol. Figure 4D demonstrates that the hydrophobic groups of JA are oriented toward the interior of the protein pocket, whereas the hydrophilic carboxyl groups are directed toward the purine groups of ATP molecules. These findings suggest that JA may serve as a potential substrate.

### 2.3. Characterization of Cis Elements in the PgJAR1 Gene Promoter

**Figure 5:**
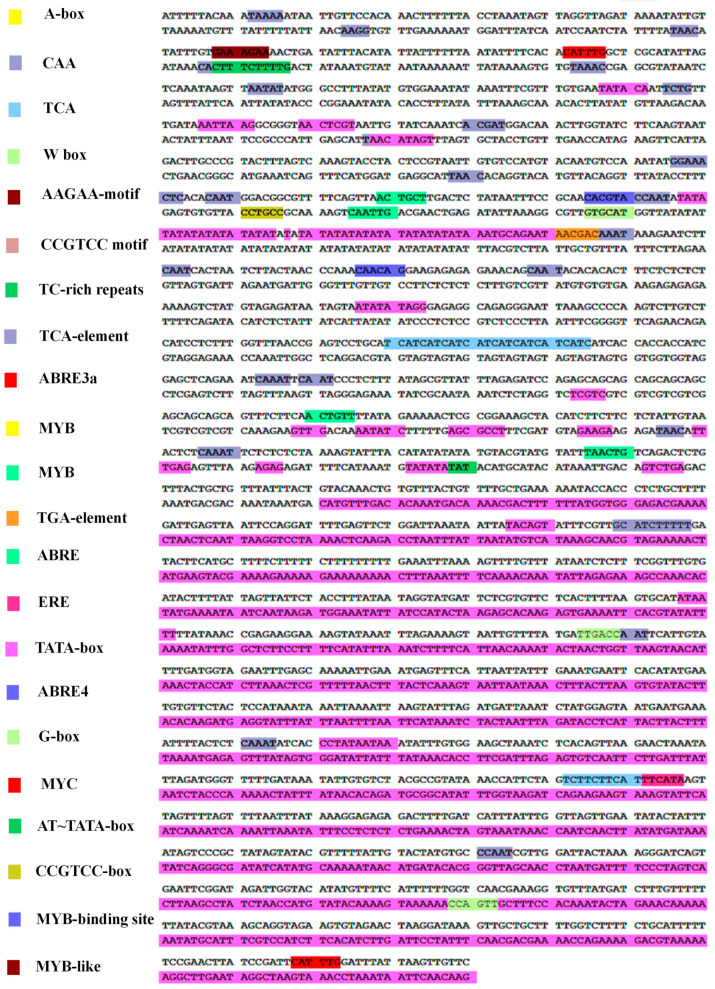
Nucleotide sequence of the *PgJAR1* promoter (*ProPgJAR1*; 1861 bp). Stress-related cis-regulatory elements are boxed and labeled with colors.

The 1861 bp upstream sequence of the initiator codon ATG in the *PgJAR1* gene was cloned and selected for the analysis of cis-acting elements in the gene promoter (*ProPgJAR1*). Prediction analysis revealed several cis-acting elements associated with stress responses (Figure 5), including the core CAAT box, which controls the frequency of transcription initiation; the TATA box, a key promoter element that binds to RNA polymerase; the CCGTCC box, a cis-acting element related to meristem expression; the G-box, which regulates light responses; A-box and TC-rich repeats, which are cis-acting elements related to defense and stress response; hormone response elements including the TGA element, ERE, ABA response element (ABRE), and AAGAA motif cis-regulatory elements related to auxin, ABA, MeJA, and other hormone responses; and a TCA element related to the SA response. *ProPgJAR1* also contains WRKY transcription factor binding sites (W-box), MYC transcription factor recognition sites related to the MeJA response, and MYB binding sites (MBS) involved in plant secondary metabolism.

**Figure 6:**
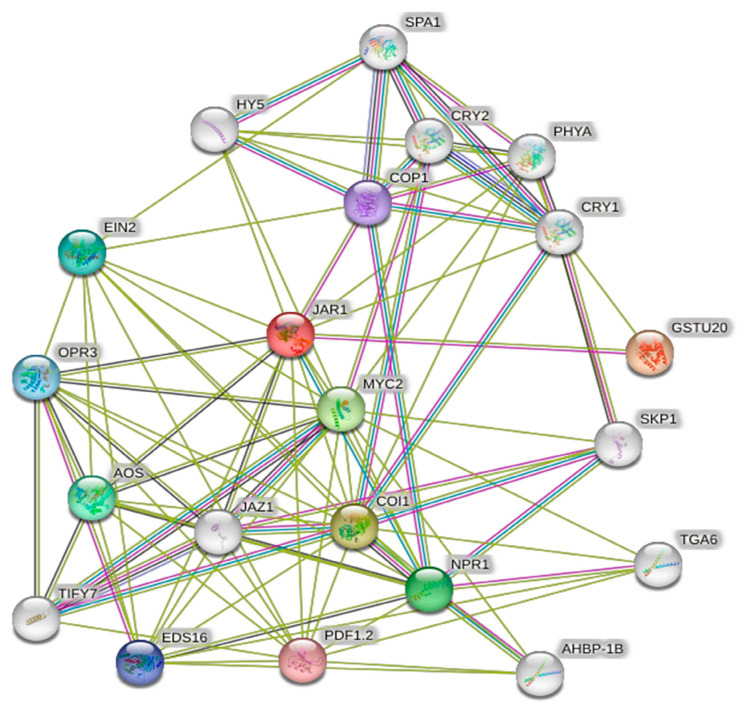
Protein–protein interaction network of the PgJAR1 protein.

The interaction network of the PgJAR1 protein in ginseng was constructed based on the interaction relationships of homologous JAR proteins from *A. thaliana* (Figure 6). Examination of this network demonstrated that the PgJAR1 protein interacts with other proteins, including OPR, AOS, JAZ, TIFY, COI1, and MYC, which play roles in the JA biosynthesis signaling pathway. Among these proteins, AOS and OPR are critical enzymes that function downstream in the JA biosynthetic pathway. AOS catalyzes the conversion of fatty acid hydroperoxides into allene oxides, which are then transformed into JA through subsequent enzymatic reactions. OPR catalyzes the reduction of 12-oxo-phytodienoic acid (OPDA) to JA, thereby playing a significant role in the regulation of jasmonate levels within the plant. The interplay between AOS and OPR is essential for the proper synthesis of and signaling by JAs, which are involved in various physiological processes, including plant defense mechanisms against herbivores and pathogens. Among the proteins, JAZ, TIFY, COI1, and MYC are integral to JA signaling; JAZ and TIFY serve as transcriptional repressors, COI1 constitutes a part of the JA receptor complex, and MYC functions as a transcriptional activator of JA-responsive genes. The interactions between PgJAR1 and these proteins imply a potential regulatory role for PgJAR1 in the JA signaling pathway. This regulation may occur through mechanisms such as modulating the stability of JAZ proteins or interacting with COI1 to influence JA signal transduction. This finding offers significant insights for further exploration of the specific roles of PgJAR1 within the plant JA signaling pathway.

### 2.4. Expression Pattern of PgJAR1 in P. ginseng

**Figure 7:**
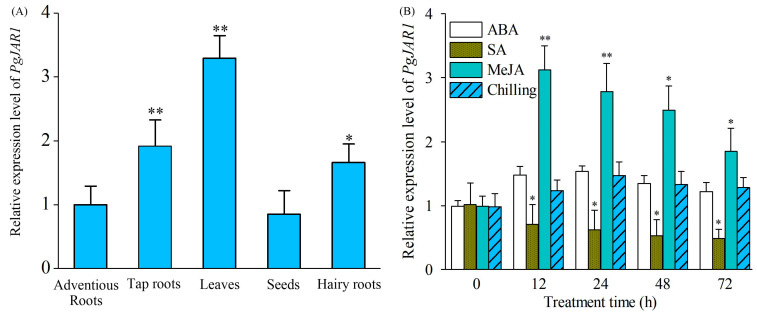
Expression pattern of *PgJAR1* in ginseng. (**A**) *PgJAR1* gene expression levels in various tissues and hairy roots of 4-year-old *P. ginseng*. The expression level of *PgJAR1* in adventitious roots was used as the control. (**B**) Differential expression patterns of *PgJAR1* in ginseng hairy roots following various stress treatments. The expression level of *PgJAR1* in hairy roots was measured at 0 h after treatment as a control. The differences between the samples and the control are statistically significant (* *p* < 0.05; ** *p* < 0.01).

The expression pattern of the *PgJAR1* gene was analyzed in various ginseng tissues, including taproots, adventitious roots, leaves, seeds, and hairy roots. The results indicated that the expression level of the *PgJAR1* gene was highest in leaves, exhibiting a level 3.29 times greater than that observed in adventitious roots. This was followed by taproots, while the expression levels in hairy roots and seeds were comparatively lower. Notably, the expression levels of the *PgJAR1* gene in leaves, taproots, and hairy roots demonstrated a statistically significant difference (*p* < 0.05 and *p* < 0.01) when compared with that in adventitious roots. There was no significant change in the expression level in seeds (Figure 7A).

Treatments involving SA, ABA, chilling (4 °C), and MeJA were carried out on the ginseng hairy roots cultured for a duration of 21 days. Post-treatment, samples were collected at intervals of 0, 12, 24, 48, and 72 h, and the expression level of the *PgJAR1* gene was subsequently analyzed using quantitative reverse transcription PCR (qRT-PCR) (Figure 7B). The findings indicated that MeJA treatment resulted in an immediate upregulation of *PgJAR1* gene expression, which peaked at 3.12-fold relative to the level at 12 h post-treatment. This elevated expression then gradually declined to 1.85-fold at 72 h, maintaining a higher expression level compared to the control (0 h). In comparison with the effects of MeJA, SA exerts a significant inhibitory influence on the *PgJAR1* gene, reducing its expression to 0.71, 0.62, 0.53, and 0.49 times the control level within 12 to 72 h post-treatment. Although ABA stress is a crucial inducer for the expression of numerous defense response-related genes, the *PgJAR1* gene does not exhibit a noticeable response to ABA. Given that ginseng is a shade-loving and cold-region medicinal plant, it was subjected to low-temperature stress at 4 °C. The findings indicate that the expression of the *PgJAR1* gene remains unaffected by this temperature stress.

### 2.5. Enzymatic Production of JA-Ile by PgJAR1

**Figure 8:**
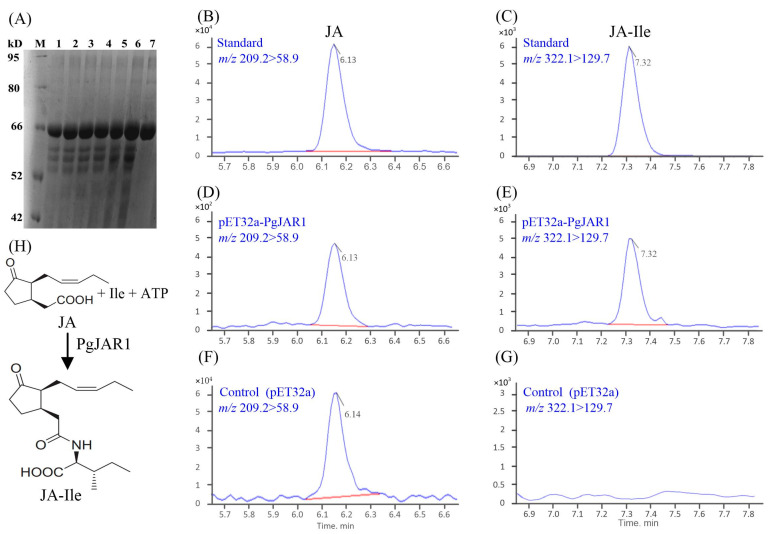
LC-MS/MS analysis of metabolites resulting from JA catalysis by recombinant PgJAR1. (**A**) The recombinant PgJAR1 protein was expressed in *E. coli* BL21 induced by IPTG. (**B**,**C**) Standard JA and JA-Ile. (**D**,**E**) The catalyzed products of JA to JA-Ile by recombinant PgJAR1 and control (containing empty pET32a) were analyzed at 0 h respectively. (**F**,**G**) Extracted ion chromatograms obtained from the catalyzed products of JA-Ile by PgJAR1 and the control for 48 h, respectively. (**H**) Proposed catalytic pathway of JA to JA-Ile by PgJAR1.

The activity of the JAR1 enzyme is crucial for the transduction of JA signals in plants. Enzymatic production of JA-Ile by JAR1 is a crucial process in the JA signaling pathway, which plays a significant role in plant defense and development. To assess the enzymatic activity of PgJAR1, *Escherichia coli* BL21 cells producing recombinant PgJAR1 were incubated with JA as the main substrate, along with ATP and Ile as co-substrates. Subsequently, all the samples were collected from the supernatant and analyzed by liquid chromatography–tandem mass spectrometry (LC-MS/MS). The resulting analytical data unequivocally demonstrated that PgJAR1 facilitates the conjugation of JA to isoleucine (Ile) by utilizing ATP and Ile within the *E. coli* system (Figure 8). To examine the substrate specificity of PgJAR1, the conjugation of JA with various amino acids was analyzed. The analysis revealed no detectable peaks corresponding to conjugates of JA with alanine (Ala), leucine (Leu), phenylalanine (Phe), or valine (Val) in the culture supernatant of *E. coli* expressing PgJAR1 (Supplementary Figure S2). Consequently, it is hypothesized that PgJAR1 exhibits a preferential specificity for isoleucine in its conjugation with JA.

### 2.6. PgJAR1-Mediated JA Biosynthesis and Biosynthesis Enzyme Gene Expression

**Figure 9:**
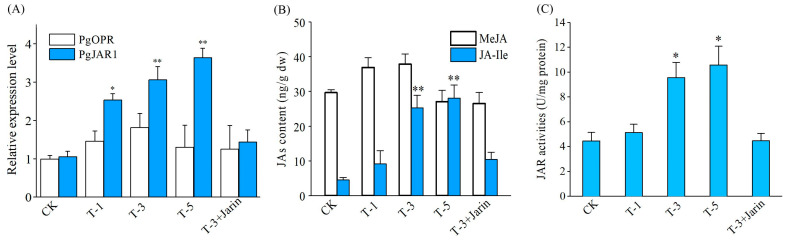
JA accumulation and the expression of key enzyme genes involved in JA biosynthesis in ginseng leaves following transient overexpression of the *PgJAR1* gene. (**A**) Expression levels of key enzyme genes in the JA biosynthesis pathway in ginseng leaves after transient overexpression of the *PgJAR1* for 1, 3, and 5 days following *Agrobacterium*-mediated infection. (**B**) MeJA and JA-Ile content and (**C**) PgJAR1 enzyme activity in ginseng leaves following transient overexpression of *PgJAR1* gene for 1, 3, and 5 days after *Agrobacterium*-mediated infection. CK represents infection by *Agrobacterium* A4 (carrying an empty vector, pCAMBIA1302); T-1, T-3, and T-5 represent infections by *Agrobacterium* A4 (carrying the vector pCAMBIA1302-PgJAR1) for 1, 3, and 5 days, respectively. T-3+Jarin-1 represents the infected ginseng leaves treated with the JA-Ile inhibitor Jarin-1 for 5 days. The differences between the treated samples and the control are statistically significant (* *p* < 0.05; ** *p* < 0.01).

In order to ascertain the potential impact of the *PgJAR1* gene on the expression levels of genes associated with the metabolic pathway of endogenous JA-Ile, which may ultimately influence JA-Ile biosynthesis, we conducted an investigation into the expression levels of the pertinent gene, *PgOPR*, in ginseng leaves subjected to transient overexpression of the *PgJAR1* gene. The ginseng leaves were infiltrated by *Agrobacterium* A4 harboring a vector pCAMBIA1302-PgJAR1 for 1, 3, and 5 days. The transient overexpression of *PgJAR1* in *P. ginseng* leaves was observed. After infiltration for 1 d, a significant increase in *PgJAR1* transcript abundance was observed in the ginseng leaves (Figure 9A). Conversely, the expression level of the upstream gene *PgOPR*, which plays a role in JA biosynthesis in ginseng, was not upregulated as a result of the transient overexpression of *PgJAR1* (Figure 9A). The content of JA-Ile, as well as the activity of the PgJAR1 enzyme, exhibited an upward trend concomitant with elevated expression levels of the *PgJAR1* gene (Figure 9B,C). These results suggest that *PgJAR1* is associated with the biosynthesis of JAs, and its overexpression in ginseng had an improvement effect on JA-Ile accumulation.

### 2.7. PgJAR1-Mediated PgSS, PgDDS, CYP, and UGT Expression and Ginsenoside Biosynthesis

**Figure 10:**
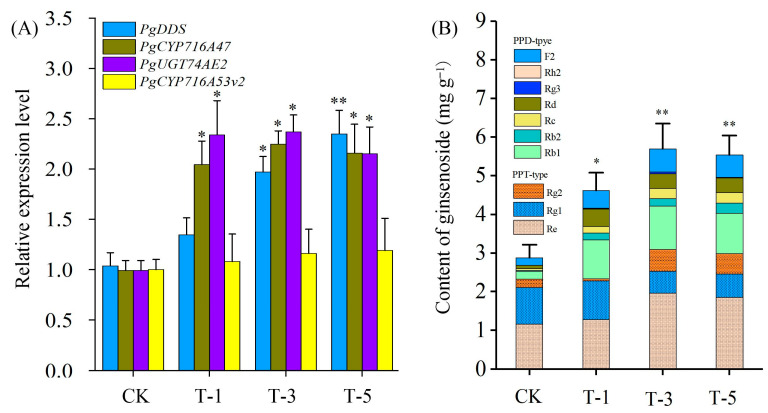
Ginsenoside accumulation and gene expression in ginseng leaves following transient overexpression of the *PgJAR1* gene. (**A**) PPD-type, PPT-type, and total ginsenoside content in ginseng leaves with transient overexpression of *PgJAR1* gene for 1, 3, and 5 days after *Agrobacterium*-mediated infection. CK represents infection by *Agrobacterium* A4 (harboring the empty vector pCAMBIA1302); T-1, T-3, and T-5 represent infection by *Agrobacterium* A4 (harboring the vector pCAMBIA1302-PgJAR1) for 1, 3, and 5 days, respectively. (**B**) Expression level of key enzyme genes of ginsenoside biosynthesis in ginseng leaves with transient overexpression of the *PgJAR1* gene for 1, 3, and 5 days after *Agrobacterium*-mediated infection. The differences between the treated samples and control are statistically significant (* *p* < 0.05; ** *p* < 0.01).

To further elucidate the impact of *PgJAR1* on ginsenoside biosynthesis, the expression levels of *PgDDS*, *PgCYP716A47*, *PgUGT74AE2,* and *PgCYP716A53v2* were analyzed following the transient overexpression of the *PgJAR1* gene in ginseng leaves. As shown in Figure 10A, three genes associated with the synthesis of PPD-type ginsenosides exhibited upregulation, whereas the expression level of *PgCYP716A53v2* gene, which is implicated in the biosynthesis of PPT-type ginsenosides, remained relatively unchanged. The JA-Ile content and expression level of *PgJAR1* were higher at T-5 compared with T-3, while the expression of ginsenoside synthase genes and the ginsenoside content were not at their peak and were slightly lower than at T-3 (Figure 10B). Notably, the expression levels of *PgDDS*, *PgCYP716A47*, and *PgUGT94Q2* reached their peak when *PgJAR1* was maintained at a relatively high level (T-3), showing increases of 1.97-, 2.24-, and 2.36-fold, respectively, compared with the control. In comparison with the control, the total ginsenoside content exhibited an increase of 1.61-, 1.98-, and 1.93-fold at T-1, T-3, and T-5, respectively. However, PPT-type ginsenosides did not demonstrate significant changes, whereas PPD-type ginsenosides increased by 4.10-, 4.67-, and 4.58-fold, respectively. These findings suggest that *PgJAR1* is particularly effective in augmenting PPD-type ginsenosides. In the aforementioned research results, higher levels of endogenous JA-Ile (compared with T-5) did not demonstrate a better effect in promoting ginsenoside accumulation. Furthermore, our findings indicate that elevated concentrations of JA-Ile suppress cell growth. The effects of varying JA-Ile concentrations on root elongation, biomass, and the growth ratio of ginseng hairy roots are comprehensively presented in Supplementary Table S3. With a JA-Ile concentration of zero serving as the control, we observed that at a concentration of 20 μmol L^−1^, root elongation, biomass, and the growth ratio (GR) were reduced to 0.9-, 0.86-, and 0.88-fold compared with their respective control values. Our results further suggest that the accumulation of ginsenosides induced by *PgJAR1* is mediated through the activation of JA-Ile, which plays a regulatory role in ginsenoside biosynthesis.

## 3. Discussion

In plants, the *GH3* gene family contains multiple types of genes, of which *JAR* is an important member. *JAR* plays a crucial role in plant growth, development, and stress responses [44]. Although several *JAR* genes have been cloned and functionally characterized in model plants, their roles in medicinal plants remain largely unclear [26,45]. In this study, we identified four homologous *JAR* genes in the MeJA-induced transcriptomic database of *P*. *ginseng,* designated as *PgJAR1, PgJAR2, PgJAR3, and PgJAR4*. *PgGH3* genes were found on 13 chromosomes, with the four *PgJAR* genes specifically located on chromosomes 7, 8, 15, and 16, while no GH3 genes were detected on the remaining 11 chromosomes, indicating an uneven distribution of *PgJAR* genes. Sequence alignment analysis revealed significant differences in DNA sequence identity among these four *JAR* homologs. These differences in sequence identity may reflect evolutionary adaptations to specific regulatory or environmental challenges in *P. ginseng*, potentially leading to functional diversification among the *PgJAR* genes. These *PgJARs* also exhibited a close phylogenetic relationship to well-characterized JA-Ile synthase genes, including *AtJAR1* from *A*. *thaliana* and *OsJAR1* and *OsJAR2* from *O*. *sativa* [27,38,46]. PgJAR1 shares high sequence homology with JAR orthologs from other plant species, such as AtJAR1 from *A. thaliana*, SlJAR1 from *S. lycopersicum,* and CsJAR4 from *Camellia sinensis*.

Structurally, PgJAR1 contains all the characteristic elements of the GH3 enzyme family, including an N-terminal and a C-terminal domain typical of the adenylating firefly luciferase, which belongs to the ANL enzyme superfamily [47]. The C-terminal domain consists of a single four-stranded beta-sheet bracketed by two alpha-helices on each side, which is similar to that found in AtGH3.11/AtJAR1 [38]. Comparative analysis of the 3D structures of PgJAR1 and AtGH3.11/AtJAR1 revealed a striking conformational similarity, further supporting the functional conservation of these proteins. Molecular docking studies suggest that PgJAR1 likely utilizes JA as a substrate, which is rapidly converted to JA-Ile following conjugation. Key amino acid residues, such as W334 (W336 in AtJAR1), L116 (L117), T120 (T121), F124 (F125), T164 (T166), and V167 (V169) are conserved between PgJAR1 and AtJAR1. These residues contribute to the formation of a hydrophobic pocket that encloses the cyclopentane ring of the substrate. Additionally, residues T164 (T166), T220 (V222), F221 (F223), and I302 (I304) are involved in forming hydrogen bonds with the substrate (Supplementary Figure S3). These structural similarities further corroborate the hypothesis that PgJAR1 is essential for the catalysis of JA to JA-Ile [38,48].

The phylogenetic proximity of *PgJAR1* to other *JAR* genes suggests a conserved role in JA-Ile biosynthesis, a critical hormone conjugate known to regulate plant defense and growth responses [48]. For instance, in tomato plants, the production of (+)-7-iso-JA-Ile is triggered solely by wounding, and in *SlJAR1*-RNAi lines, JA-Ile is reduced by 50–75%, affirming the importance of *SlJAR1* in JA-Ile biosynthesis. Promoter analysis of *PgJAR1* revealed the presence of various cis-regulatory elements associated with plant growth, development, light responses, hormone signaling, and stress adaptation, further highlighting its potential regulatory roles [44,49].

The interaction network analysis revealed that the PgJAR1 protein interacts with several proteins involved in the JA signaling pathway, including allene oxide synthase (AOS), 12-oxo-phytodienoate reductase (OPR), JAZ, TIFY, COI1, and MYC [48,50]. JAs such as JA, MeJA, and JA-Ile are known to exhibit heightened sensitivity to environmental stimuli [51]. The biosynthesis of JAs in plants is regulated by a series of enzymes, including lipoxygenase (LOX), AOS, OPR, and JAR [50]. For example, in *A. thaliana*, the AtJAR1 enzyme catalyzes the formation of JA-Ile, which is crucial for activating defense responses [52]. In the absence of JA-Ile, JAZ proteins act as negative regulators of the JA signaling pathway by inhibiting the activity of MYC transcription factors. However, upon JA-Ile perception, COI1 forms a complex with JAZ proteins, leading to their degradation and the subsequent activation of MYC transcription factors. This activation promotes the expression of JA-responsive genes [17,20,53,54]. In *Salvia miltiorrhiza*, specific JAZ proteins have been shown to repress the biosynthesis of tanshinones and salvianolic acids [21]. Furthermore, the interaction between MYC and JAZ proteins is critical for the transcriptional activation of JA-responsive genes, highlighting the central role of these components in the JA signaling network [55,56].

*PgJAR1* exhibits a tissue-specific expression pattern, with the highest level observed in ginseng leaves. This is consistent with the role of JAs in mediating leaf-associated defense mechanisms against herbivory and pathogens [57]. The upregulation of *PgJAR1* following MeJA treatment further supports its responsiveness to JA signaling and its role in the JA-Ile biosynthetic pathway. Moderate and transient overexpression of *PgJAR1* in ginseng leaves resulted in a significant increase in both JA-Ile and ginsenoside accumulation. This finding aligns with previous studies demonstrating the role of JAs in regulating the production of secondary metabolites, including ginsenosides, which are crucial pharmacologically active compounds in ginseng [58]. However, excessive JA-Ile levels were found to inhibit cellular growth, leading to reduced ginsenoside production. This observation is consistent with studies showing that high JA levels can negatively impact plant growth, suggesting a trade-off between defense responses and growth promotion [41,59,60,61]. Additionally, SA has been shown to modulate plant defense responses by antagonizing the JA signaling pathway [62]. In this study, SA significantly suppressed the expression of the *PgJAR1* gene, further illustrating the complex interplay between JA biosynthesis and ginsenoside production [63,64].

Numerous studies have demonstrated that JAs are more abundant in leaves compared with other plant tissues. For instance, one study effectively quantified JAs in plants and confirmed that their concentrations are generally higher in leaves than in roots, stems, or flowers [65]. This distribution pattern may be linked to the role of JAs in mediating leaf-specific defense mechanisms. The application of exogenous JA has been shown to induce the redistribution of defensive compounds within leaves, suggesting that JAs play a key role in plant stress responses [66]. Similarly, a systematic evaluation of ginsenoside content in various tissues of *P. ginseng* aged from 1 to 13 years revealed that leaves consistently contain higher levels of ginsenosides compared with rhizomes and main roots [67]. The elevated levels of both JAs and ginsenosides in leaves may reflect their synergistic roles in plant defense mechanisms and secondary metabolic activities.

Ginsenosides, a class of triterpene saponins, are biosynthesized through a highly regulated enzymatic pathway. For example, PgSS catalyzes the condensation of farnesyl pyrophosphate to form squalene, while PgSE mediates the epoxidation of squalene to produce 2,3-oxidosqualene. This intermediate is then converted by PgDDS into dammarenediol-II, a key precursor for various ginsenosides [58,68,69]. In addition to PgDDS, cytochrome P450 enzymes (CYPs) and UDP-glycosyltransferases (UGTs) play critical roles in the structural diversification of ginsenosides. For instance, CYP716A47 catalyzes the oxidation of dammarenediol-II to form protopanaxadiol, a major intermediate in ginsenoside biosynthesis [70]. Meanwhile, PgUGT74AE2 transfers a glucose moiety from UDP-glucose to protopanaxadiol and compound K, yielding the ginsenosides Rh2 and F2, respectively. Similarly, PgUGT94Q2 facilitates the glycosylation of Rh2 and F2 to produce Rg3 and Rd [71]. Another key enzyme, CYP716A53v2, is essential for the conversion of protopanaxadiol to protopanaxatriol, a critical step in the biosynthesis of protopanaxatriol-type ginsenosides such as Rh1, Rg1, and Re [72,73]. The biosynthesis of ginsenosides is tightly regulated by JAs, environmental factors, and stress conditions. Research has shown that JAs significantly influence the expression of genes involved in ginsenoside biosynthesis, such as *PgDDS*, *PgCYP716A47*, and *PgUGT94Q2* [74]. Overexpression of the *PgJAR1* gene in ginseng leaves enhances the biosynthesis of endogenous JA-Ile, which, in turn, upregulates these genes and promotes the accumulation of PPD-type ginsenosides. These findings highlight the central role of JA-Ile in coordinating ginsenoside biosynthesis and stress responses.

From a broader perspective, these results contribute to our understanding of the intricate regulatory networks governing plant responses to environmental stress and growth regulation [59]. The expression of PgJAR1 and the subsequent production of JA-Ile appear to play a pivotal role in balancing defense responses and secondary metabolism. This suggests that modulating endogenous JA-Ile levels could be a promising strategy to optimize ginseng cultivation and to enhance its medicinal properties. Future research should focus on elucidating the molecular mechanisms by which JA-Ile influences cellular proliferation and ginsenoside biosynthesis. This could involve exploring the crosstalk between JA signaling and other phytohormone pathways, such as auxin and cytokinin, which are known to regulate plant growth and development. Additionally, the development of genetic tools to precisely control *PgJAR1* expression and JA-Ile levels in ginseng plants could provide a means to fine-tune ginsenoside production and to improve plant resilience to environmental stress.

## 4. Materials and Methods

### 4.1. Chemicals, Materials, and Treatment

The ginsenoside standards Rb1, Rb2, Rc, Rd, Re, and Rg1, procured from Chengdu Herbpurify (Chengdu, China), were of chromatographic grade, while all other reagents utilized were of analytical grade. Hairy roots were induced by *A. rhizogenes* A4 from four-year-old fresh ginseng (*P. ginseng* C.A. Meyer), which was collected from Fusong County, Jinlin Province, China [75]. The ginseng hairy roots were cultured in flasks containing ½ MS liquid medium at 25 °C with shaking at 110 rpm. After a preculture of 21 d, the hairy roots were treated with the corresponding chemicals at different concentrations. After the treatment, the hairy roots were collected and frozen in liquid nitrogen for further use.

### 4.2. Chromosome Localization Analysis of Ginseng GH3 Genes

The GH3 genes in *P. ginseng* were identified using the UniGene database derived from previous studies. To determine their genomic distribution, the GH3 gene sequences were compared against the ginseng genome. The analysis employed stringent criteria, including 100% sequence identity and a coverage length of ≥400 bp, to accurately assign the gene family members to their respective genomic locations. The chromosomal positions of the transcripts were visualized using the MG2C online tool (http://mg2c.iask.in/mg2c_v2.1/index.html; accessed on 16 February 2025), which facilitated the mapping and interpretation of the spatial distribution of GH3 genes across the ginseng genome.

### 4.3. Gene Cloning and Bioinformatic Analysis

The sequence information of *PgJAR1* was obtained from the transcriptomic data of ginseng hairy roots under 100 μmol L^−1^ MeJA induction for 12–72 h. Genomic DNA was extracted from fresh ginseng roots using a Plant DNA Kit (Omega). Total RNA was isolated from ginseng hairy roots using the Plant RNA Kit (Omega, Doraville, GA, USA). The HiFiScript gDNA Removal RT MasterMix (CoWin Biotech Co. Ltd., Beijing, China) was used to remove the genomic DNA and to synthesize the first-strand cDNA. The Super Pfx DNA Polymerase (CoWin Biotech Co. Ltd., Beijing, China) was used to obtain full-length cDNA of *PgJAR1* with the cloning primers (Supplementary Table S4). The cDNAs were inserted into pEASY-Blunt (TransGen Biotech, Beijing, China) and verified through sequencing. The ORF analysis and amino acid sequence comparison were performed using ORF finder (http://www.ncbi.nlm.nih.gov/orffinder/; accessed on 8 July 2024). The amino acid sequence comparison analysis of JARs was performed using BLAST-P (Protein-basic local alignment search tool) (https://blast.ncbi.nlm.nih.gov/Blast.cgi; accessed on 12 July 2024). The protein length, molecular weight, and isoelectric point (pI) were determined utilizing the ProtParam tool (https://web.expasy.org/protparam/; accessed on 12 July 2024). The Conserved Domain Database (CCD) provided by NCBI (http://www.ncbi.nlm.nih.gov/Structure/cdd/wrpsb.cgi; accessed on 8 September 2024) was utilized with predefined parameters to assess the accuracy of the PgJAR1 sequence. Alignments of the deduced PgJAR1 protein were executed using the Clustal W version 1.8 softwar. Phylogenetic analysis of the deduced amino acid sequences was carried out using MEGA 5.1 with the neighbor-joining method. To evaluate the robustness of the phylogenetic tree nodes, a bootstrap analysis with 1000 replicates was performed. The evolutionary distances were represented through a neighbor-joining tree, calculated using the *ρ*-distance model. The protein sequence alignment incorporated a gap opening penalty of 10 and a gap extension penalty of 0.2. The PlantCare database (http://bioinformatics.psb.ugent.be/webtools/plantcare/html; accessed on 11 October 2024) was used to analyze the cis-regulatory elements of the *PgJAR1* gene. Additionally, an investigation into protein interactions was carried out utilizing the STRING database (https://cn.string-db.org/; accessed on 1 October 2024), which is specifically designed for protein interaction analyses.

### 4.4. Homology Modeling and Molecular Docking

The template crystal structure for PgJAR1 was verified through BLAST and downloaded from the RCSB Protein Data Bank (PDB code: 5ECI). Homology modeling was conducted using Yasara v16 [76]. Models were built based on a target–template alignment using ProMod3 version 1.0.0 [77]. The final stable geometry of the resulting model was confirmed and regularized using a force field. AutoDock 4.2 [78] was used for the molecular docking of PgJAR1 with the candidate substrate JA and ATP for the analysis of binding affinity and binding sites. The structure of JA and ATP were acquired from PubChem [79] (ID: 5281166, 5937) and converted to 3D by Avogadro through energy minimization. Subsequently, a conformational search was conducted to verify the stable geometry for the docking preprocessing. The binding site was identified within a beta-sheet barrel, which was encased by an alpha-helix. The amino acid residues involved in the reaction were situated within a docking box, centrally positioned within the barrel, with dimensions of 50 × 50 × 50 Å. All docked poses of JA were ranked by binding energy, and the threshold of cluster analysis was set at 5 angstroms. Any parameters not mentioned above were set to default values.

### 4.5. Prokaryotic Expression of PgJAR1 and Enzyme Assay

The full-length cDNA of *PgJAR1* was cloned using prokaryotic expression primers (Supplementary Table S4) and ligated into the pET23a(+) vector. After ligation, the recombinant pET23a-PgJAR1 was expressed in *E. coli* BL21 (DE3). Bacteria harboring the empty pET23a vector were used as a control. PgJAR1 activity was assayed according to method of [46], with slight modifications. The transformed *E. coli* BL21 (DE3) cells carrying pET23a-PgJAR1 or pET23a were cultured overnight at 37 °C in 10 mL of LB medium with 100 mg mL^−1^ ampicillin. A portion of the culture was cultured overnight at 37 °C in LB medium containing 100 μg mL^−1^ ampicillin until the OD600 reached 0.2, and then 1 mM IPTG was added at 37 °C for 5 h to induce protein production. Subsequently, 0.2 mM JA was added to the culture. The co-culture was performed at 37 °C for another 3 h with shaking. The presence of JA-Ile in the culture was determined using the HPLC-MS/MS as described in Section 4.6.

### 4.6. Transient Expression Analysis of PgJAR1

In order to transiently overexpress *PgJAR1* in ginseng leaves, the *PgJAR1* gene was cloned using transient expression primers (Supplementary Table S4) and inserted into pCAMBIA1302 at the *BgI* II site. Then, the *Agrobacterium rhizogenes* A4 strain harboring pCAMBIA1302-PgJAR1 or pCAMBIA1302 (control) was used for transformation. The A4 cells were cultured at 30 °C overnight on a shaker at 250 rpm in LB medium containing 40 μmol L^−1^ acetosyringone (AS) and 0.01 M MES. The bacteria were collected by centrifugation and resuspended in 0.2 mM AS and 10 mM MgCl_2_. The A4 suspension was injected into the lower epidermis of ginseng leaves of seedlings (about 2 months) using a sterile syringe [80]. The injected ginseng plants were grown at 25 °C for 1–5 days, and then the infiltrated leaves were cut for subsequent study.

### 4.7. Measurement of JA-Ile and MeJA Levels

JA-Ile and MeJA levels were determined by Convinced-test Technology Co., Ltd. (Nanjing, China) using HPLC-MS/MS. Approximately 0.5 g of fresh ginseng tissue was ground immediately to a powder after liquid nitrogen treatment in a pre-cooled mortar. JA-Ile was extracted using 5 mL of isopropanol at 4 °C for 60 min. The extracts were filtrated with filter paper and extracted with an equal volume of dichloromethane. The extracts were shaken gently for 30 min and then centrifuged at 12,000 rpm for 5 min at 4 °C. After evaporating the dichloromethane layer under N_2_, the samples were dissolved with methanol (0.1% formic acid) and filtered using a 0.22 µm membrane for HPLC-MS/MS analysis. JA-Ile and MeJA were analyzed using an Agilent (Santa Clara, CA, USA) ZORBAX SB-C_18_ column (3.5 μm, 2.1 mm × 150 mm), with elutions performed with solvent A, consisting of methanol/0.1% formic acid, and solvent B, consisting of ultrapure water/0.1% formic acid, as the mobile phase at 45:55 (*v/v*). The spray voltage was 4500 V; the pressure of the aux gas, nebulizer, and air curtain was 70, 65, and 15 psi, respectively; and the atomizing temperature was 400 °C.

### 4.8. Determination of Ginsenoside Content

The extraction and analysis of ginsenosides were performed using methanol and the LC-MS method, with slight modifications [58]. Ginseng tissues (hairy roots or leaves) were collected and washed three times with purified water. These were dried to a constant weight at 60 °C for 24–48 h. The samples were ground to powder and soaked in 80% methanol at 60 °C with 2 h of ultrasonic treatment. Then, the extracts were filtrated with filter paper, washed with ether, and extracted with n-butanol. After evaporating the butanol layer, the samples were dissolved with methanol and filtered through a 0.22 µm membrane for analysis. The total ginsenoside content was analyzed using an Agilent 6420 triple quadrupole mass spectrometer. LC-MS analyses were performed by a full scan in the negative-ion mode. High-purity nitrogen was used as the drying gas (11 L/min) and nebulizer gas (15 psi) at a spray voltage of 4000 V. The atomizing temperature was 300 °C.

### 4.9. Gene Expression Analysis of Key Enzymes of JA and Ginsenoside Biosynthesis

To evaluate the expression patterns of key enzyme genes of JA and ginsenoside biosynthesis, six genes, including *PgOPR*, *PgJAR1*, *PgDDS*, *PgCYP716A47*, *PgUGT74AE2,* and *PgCYP716A53v2*, were selected from the major pathways and validated by qRT-PCR on the Mini Opticon real-time system (Bio-Rad, Hercules, CA, USA). Ginseng hairy roots or leaves were collected and freeze-dried in liquid nitrogen. Total RNA was extracted from ground powder according to the manufacturer’s instructions (Plant RNA Kit; Omega, Doraville, GA, USA). Total RNA was reverse-transcribed into cDNA using the SuperRT One Step RT-PCR Kit (CoWin Biotech Co. Ltd., Beijing, China). The primers were designed using NCBI Primer-BLAST tools. To ensure primer specificity, the sequences of *PgJAR1* were aligned with those of *PgJAR2*, *PgJAR3*, and *PgJAR4*. The primer sequences are presented in Supplementary Table S5. The gene expression analysis was performed using the UltraSYBR One Step RT-qPCR Kit (CoWin Biotech Co. Ltd., Beijing, China). The expression levels were calculated using the 2^−ΔΔCt^ formula after normalization with *β-actin* [49].

### 4.10. Statistical Analysis

SPSS (version 17.0, Chicago, IL, USA) was used to calculate the data and to perform the statistical analyses. Each experiment was performed with three technical and three biological replicates. All the data were statistically analyzed, and all the values are presented as the mean value with standard deviation (SD). The results were statistically analyzed using one-way ANOVA (analysis of variance) with the Duncan test (*p* < 0.05).

## 5. Conclusions

**Figure 11:**
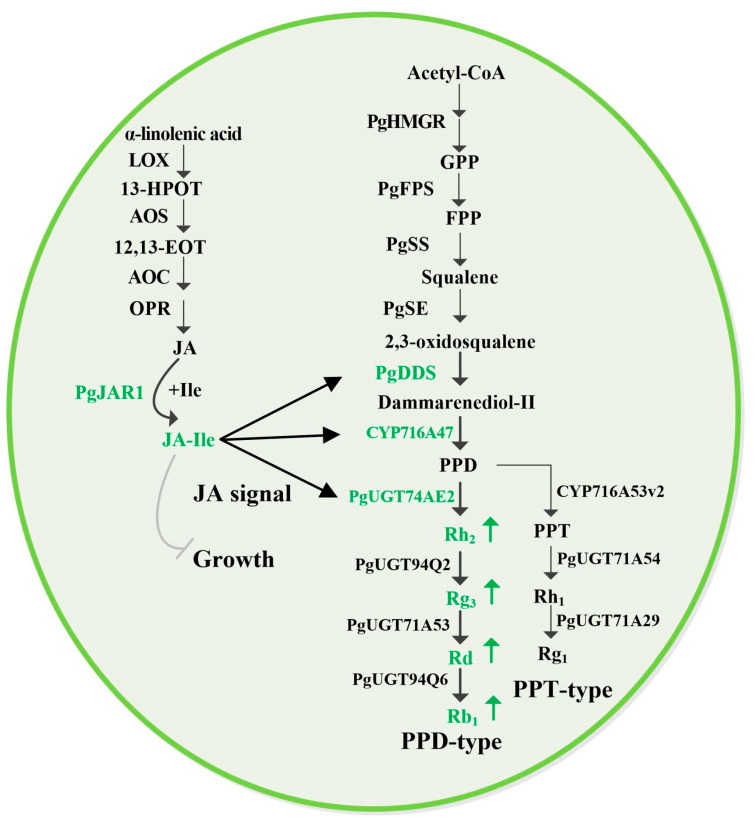
Proposed model for the regulation of ginsenoside biosynthesis following PgJAR1-mediated JA signaling pathways. (LOX, lipoxygenase; AOS, allene oxide synthase; AOC, allene oxide cyclase; OPR, OPDA reductase 12-oxophyto-dienoic acid reductase; JAR1, JA-Ile synthase; HMGR, 3-hydroxy-3-methylglutaryl-CoA reductase; FPS, farnesyl diphosphate synthase; SS, squalene synthase; SE, squalene epoxidase; CYP, cytochrome P450 reductase; DDS, dammarenediol-II synthase; UGT, UDP-glycosyltransferase including PgUGT74AE2, PgUGT94Q2, PgUGT94Q6, PgUGT71A53, PgUGT71A54, and PgUGT71A29; PPD, protopanaxadiol; PPT, protopanaxatriol). The arrows indicate positive regulation, whereas the bars denote inhibition.

In this study, the *PgJAR1* gene was successfully cloned from *P. ginseng*. It consists of three introns and four exons and is located on chromosome 7. The promoter of *ProPgJAR1* possesses cis-regulatory elements associated with plant growth, development, light responses, hormone responses, and stress adaptation. The interaction network demonstrated that the PgJAR1 protein interacts with proteins such as OPR, AOS, JAZ, TIFY, COI1, and MYC, which play roles in the biosynthesis of JAs and the JA signaling pathway. The expression pattern of *PgJAR1* is specific in different tissues, exhibiting the highest expression levels in leaves, and is upregulated by MeJA and inhibited by SA. PgJAR1 specifically promotes the ATP- and Ile-dependent conjugation of JA to Ile in the *E. coli* system, confirming its enzymatic role in JA-Ile biosynthesis. The moderate transient overexpression of *PgJAR1* gene in ginseng leaves enhances the expression of *PgDDS*, *PgCYP716A47*, and *PgUGT94Q2* genes through the biosynthesis of endogenous JA-Ile, ultimately promoting the accumulation of PPD-type ginsenosides. Nevertheless, elevated levels of JA-Ile impede cellular growth, leading to a reduction in ginsenoside production. The expression of the *PgJAR1* gene and the biosynthesis of JA-Ile may play a pivotal role in balancing the plant’s response to environmental stress and growth regulation. Therefore, modulating endogenous JA-Ile levels could represent a significant strategy for augmenting ginsenoside production (Figure 11). These findings open up avenues for future research aimed at harnessing the potential of JA signaling to enhance the medicinal value of ginseng and other medicinal plants.

## Data Availability

Data are contained within the article and Supplementary Materials.

## Supplementary Materials

The following supporting information can be downloaded at: https://www.mdpi.com/article/10.3390/plants14060847/s1, Figure S1: The expression level of *PgJAR2*, *PgJAR3*, and *PgJAR4* in ginseng hairy roots following 100 μM MeJA treatment for 0, 12, 24, 48, and 72 h. Log2Fold change were calculated from FPKM (fragments per kilobase of exon model per million mapped) based on the transcriptomic data; Figure S2: LC/ESI-MS analysis of conjugates of JA and amino acid (JA-Ala, JA-Val, JA-Phe, and JA-Leu) in the culture supernatant of *E. coli* BL21 expressing PgJAR1. (A,C,E,H) Standard of JA-Ala, JA-Val, JA-Phe, and JA-Leu. No obvious peak corresponding to JA-Ala, JA-Val, JA-Phe, and JA-Leu was detected in the culture supernatant of *E. coli* BL21 expressing PgJAR1(B,D,F,I); Figure S3: Sequence comparison of residues in the binding sites of AtJAR1 from *Arabidopsis* according to the 3D structure. Numbering at the top and bottom correspond to PgJAR1 and AtJAR1, respectively. Black solid triangle across the bottom of the alignment indicate residues with side chains in the pocket; black shade indicates conservation; Table S1: Nucleotide sequence of 21 GH3 genes from ginseng; Table S2: Orthologous proteins from other species for phylogenetic tree construction; Table S3: Effect of JA-Ile concentration on the growth of ginseng hairy roots; Table S4: Cloning and expressing primers; Table S5: Primers of the selected genes verified by qRT-PCR analysis.
