# Supplementary material for: Harnessing Jasmonate Pathways: PgJAR1’s Impact on Ginsenoside Accumulation in Ginseng
Source: Plants (Basel). 2025 Mar 8;14(6):847. doi: 10.3390/plants14060847 (PMC11945057; doi:10.3390/plants14060847)
Supplement: Supplementary file 1 [file plants-14-00847-s001.zip › plants-3433326-supplementary.pdf]

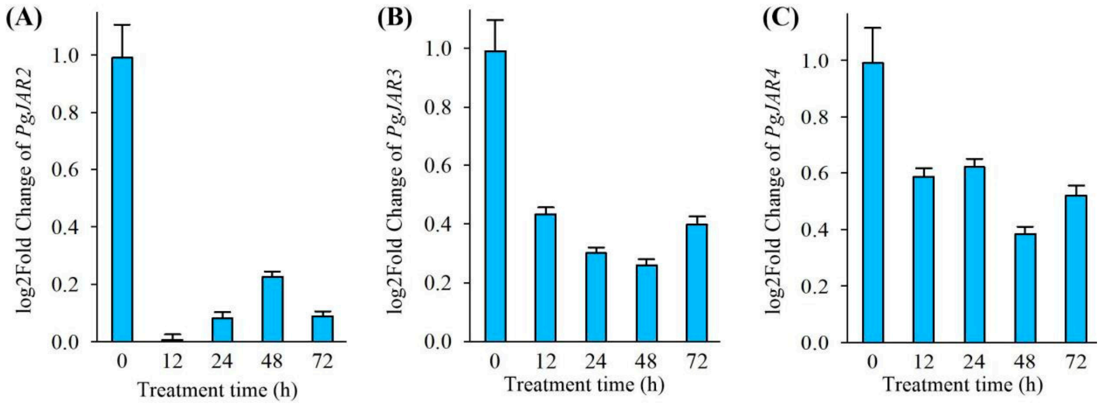

Supplementary Figure S1. The expression level of *PgJAR2*, *PgJAR3*, and *PgJAR4* in ginseng hairy roots following 100 μM MeJA treatment for 0, 12, 24, 48, and 72 h. Log2Fold change were calculated from FPKM (fragments per kilobase of exon model per million mapped) based on the transcriptomic data.

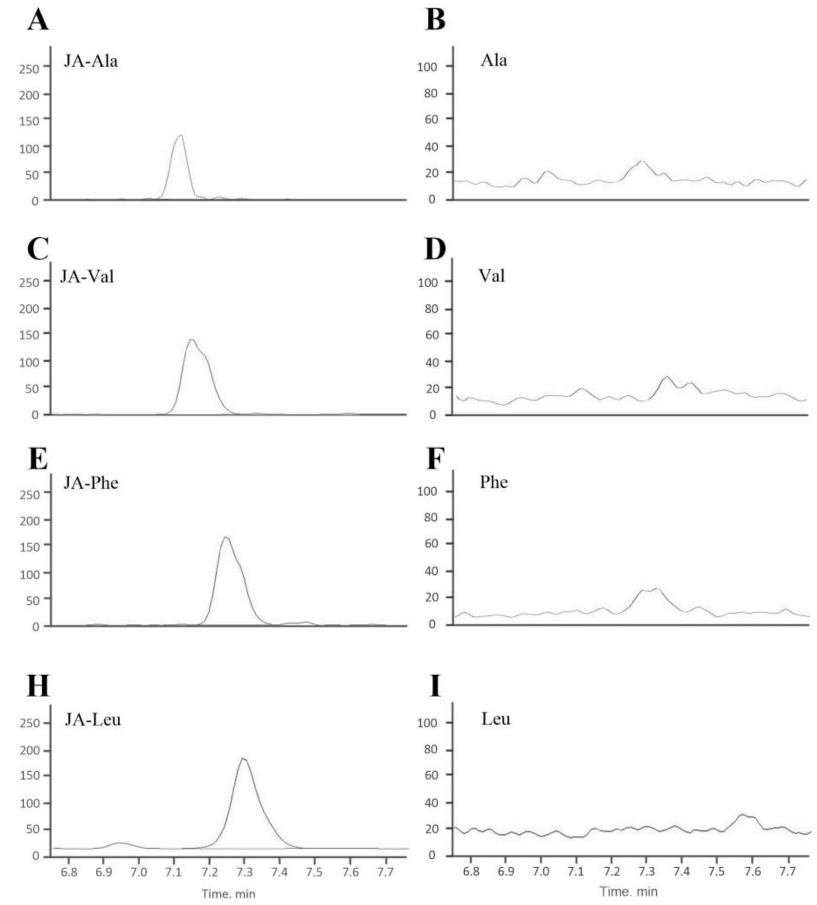

Supplementary Figure S2. LC/ESI-MS analysis of conjugates of JA and amino acid (JA-Ala, JA-Val, JA-Phe, and JA-Leu) in the culture supernatant of *E. coli* BL21 expressing PgJAR1. (**A,C,E,H**) Standard of JA-Ala, JA-Val, JA-Phe, and JA-Leu. No obvious peak corresponding to JA-Ala, JA-Val, JA-Phe, and JA-Leu was detected in the culture supernatant of *E. coli* BL21 expressing PgJAR1(**B,D,F,I**).

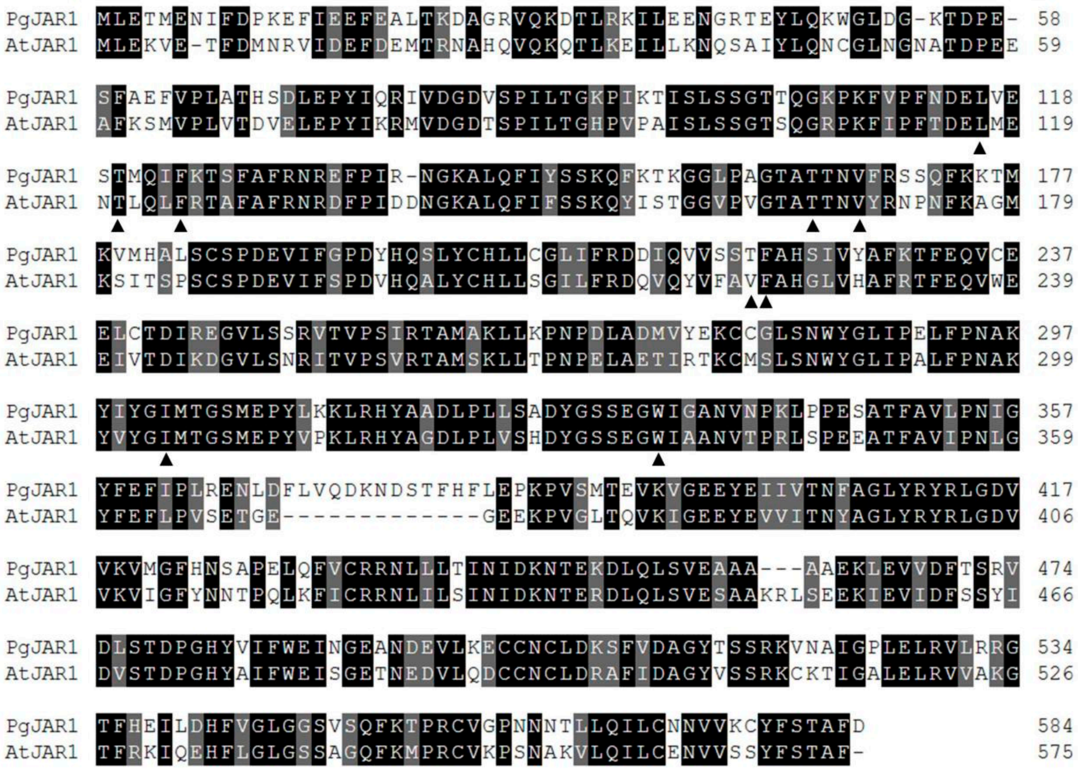

Supplementary Figure S3. Sequence comparison of residues in the binding sites of AtJAR1 from *Arabidopsis* according to the 3D structure. Numbering at the top and bottom correspond to PgJAR1 and AtJAR1, respectively. Black solid triangle across the bottom of the alignment indicate residues with side chains in the pocket; black shade indicates conservation.

Supplementary Table S1. Nucleotide sequence of 21 GH3 genes from ginseng

NO	gene_id	Sequence
1	PgJAR1 (PgGH3.6)	ATGTTGGAAACTATGGAGAATATTTTTGACCCCAAAGAATTTATAGAAGAATTTGAGGCTCTGACGAAGGATGCCGGGAGAGTTCAAAAGGATACCTTAAGGAAAATATTGGAAG<br>AAAATGGTAGAACAGAGTATTTACAGAAATGGGGTCTTGATGGAAAAACTGATCCCGAGAGTTTTGCAGAATTTGTGCCTCTTGCCACTCACAGTGATTTGGAACCTTACATTCAA<br>AGGATCGTGGATGGTGATGTTTCCCCGATTCTCACCGGAAAGCCCATCAAAACCATCTCATTAAGTTCTGGAACTACTCAGGGTAAGCCCAAGTTTGTACCTTTCAATGATGAATTA<br>GTGGAGTCCACTATGCAGATATTTAAGACATCTTTTGCCTTTAGAAACAGAGAGTTTCCTATCCGAAATGGAAAGGCCTTGCAGTTTATCTATAGCAGCAAGCAGTTCAAAACAAA<br>AGGGGGACTGCCAGCAGGAACGGCTACTACAAATGTGTTTCGTAGTTCACAATTCAAGAAAACAATGAAGGTAATGCATGCCTTGAGTTGTAGCCCGGATGAAGTGATATTTGGT<br>CCTGATTACCACCAGTCCTTGTACTGCCATCTCCTATGTGGACTTATTTTCCGGGATGACATACAAGTTGTGTCCTCTACATTTGCCCACAGCATTGTTTATGCCTTCAAGACTTTTGA<br>ACAAGTCTGTGAAGAACTCTGTACTGACATCCGAGAAGGAGTCTTGAGCAGTCGAGTTACTGTTCCATCCATCCGAACAGCTATGGCGAAACTGCTCAAGCCTAATCCTGATTTGG<br>CTGACATGGTTTACGAAAAGTGTTGTGGGTTAAGTAATTGGTATGGATTAATACCAGAGCTATTTCCCAATGCTAAGTACATATATGGTATCATGACAGGGTCAATGGAACCTTATTT<br>AAAAAAATTGAGGCACTATGCAGCGGACCTACCGCTATTGAGTGCTGATTATGGGTCTTCTGAAGGGTGGATTGGGGCAAATGTTAACCCGAAATTGCCCCCGGAGTCGGCAACT<br>TTTGCTGTGCTTCCTAATATTGGGTATTTCGAATTTATACCCCTGAGGGAGAATCTGGACTTCCTGGTTCAAGATAAAAATGATTCCACTTTCCACTTTTTAGAGCCCAAGCCAGTGA<br>GTATGACTGAAGTCAAGGTTGGCGAAGAATACGAGATCATTGTCACCAATTTCGCAGGTCTGTACAGGTATAGATTAGGAGATGTGGTTAAGGTTATGGGATTTCACAACTCGGCC<br>CCGGAACTCCAATTTGTTTGTAGGAGAAATCTTCTGCTCACCATCAACATTGATAAGAACACGGAGAAAGATTTACAGCTATCGGTTGAAGCAGCAGCAGCTGCAGAAAAACTTG<br>AAGTTGTGGATTTCACAAGCCGTGTCGATTTATCCACAGATCCCGGTCATTATGTCATCTTCTGGGAAATAAATGGCGAAGCAAATGACGAGGTTCTGAAGGAATGCTGCAATTGTT<br>TGGACAAATCATTTGTGGATGCAGGCTATACGAGCTCCCGAAAGGTGAATGCCATAGGACCACTCGAGCTCCGAGTCTTGAGGAGGGGAACTTTTCATGAGATTCTTGATCATTTT<br>GTAGGATTGGGTGGCTCTGTAAGCCAATTCAAAACTCCTCGATGCGTCGGACCCAATAATAACACACTGTTGCAGATACTATGCAACAATGTTGTCAAGTGCTACTTTAGTACTGCT<br>TTTGATTGA
2	PgJAR2 (PgGH3.18)	ATGGCGAAACTGCTCAAGCCTAATCCTGATTTGGCTGACATGGTTTATGAAAAGTGTTGTGGGTTAAGTAATTGGTATGGATTAATACCAGAGCTATTTCCCAATGCTAAGTACATAT<br>ATGGTATCATGACAGGGTCAATGGAACCTTATTTAAAAAAATTGAGGCACTATGCAGCGGACCTACCGCTACTGAGTGCTGATTATGGGTCTTCTGAAGGGTGGATTGGGGCAAAT<br>GTTAACCCGAAATTGCCCCCGGAGTCTGCAACTTTTGCTGTGCTTCCTAATATTGGGTATTTCGAATTTATACCCCTGAGGGAGAATCTGGACTTCCTGGCTCAAGATAAAAATGATT<br>CCACTTTCCACTTTTTAGAGCCCAAGCCAGTGAGTATGACTGAAGTCAAGGTTGGCGAAGAATACGAGATCATTTTCACCAATTTCTCAGGGCTGTACAGGTATAGATTAGGAGAT<br>GTGGTTAAGGTTATGGGATTTCACAACTCGACCCCGGAACTCCAATTTGTTTGTAGGAGAAATCTTCTGCTCACCATCAACATTGATAAGAACACCGAGAAAGATTTACAGCTATC<br>GGTTGAAGCAGCAACAGCTGCAGAAAAACTTGAAGTTGTGGATTTCACAAGCCGTGTGGATTTATCCACAGATCCCGGTCATTATGTCATCTTCTGGGAAATAAATGGCGAAGCA<br>AATGACGAGGTTCTGAAGGAATGCTGCAATTGTTTGGACAAATCATTTGTGGATGCAGGCTATACGAGCTCCCGAAAGGTGAATGCCATAGGACCACTCGAGCTCCGAGTCTTGA<br>GGAGGGGAACTTTTCATGAGATTCTTGATCATTTTGTAGGATTGGGTGGCTCTGTAAGCCAATTCAAAACTCCTCGATGCGTCGGACCCAATAATAACACACTGTTGCAGATACTAT<br>GCAACAATGTTGTCAAGTGCTACTTTAGTACTGCTTTTGATTGA
3	PgJAR3 (PgGH3.16)	ATGTTGGAAAAGATGGAGAAAATATTTGACCATGAAGAATTTATTGAAGAATTTGAGGCTCTGACGAAGGATGCAGGGAGAGTTCAAAAGGAGACCTTAAGGAAAATATTGGAA<br>GAAAATCGAAATACAGAATACTTACAGAAATGGGATCTTGATGGAAAAACTGATCCGGAGAGTTTTGCAAAATGTGTGCCTCTTGTCACTCACAAGGATTTGGAACCTTACATTCA<br>GAGAATCGTGGATGGTGATGTTTCCCCAATTCTCACTGGAAAACCGATAAAAACCATCTCACTAAGTTCCGGCACTACTCAAGGTAAGCCCAAGTTTGTACCTTTCAATGATGATTT<br>GGTGGAATCTACTATGCAGATATATAAGACATCTTTTGCCTTTAGAAACAGAGATTTTCCAATTCGAAATGGAAAGGCCTTACAGTTTATCTATGGTAGCAAGCAGTTTAGAACCAA<br>AGGGGGACTGCCAGCAGGAACTGCTACTACAAATGTGTATCGTAGTTCACAGTACAAGAAAACAATGAAGGCAATGCAGACCCCGTGTTGTAGCCCGGATGAAGTGATATTTGGT<br>CCTGACTTCCACCAATCCTTGTATTGCCATCTCCTATGTGGACTTATTTCCCGGGATGACGTTCAAGTTGTGTCCTCTACATTTGCTCACAGCATTGTTCATGGCTTCAGGACTTTTGA<br>ACAAGTTTGGGAAGAACTCTGTACTGATATCCGAGAAGGAGTCCTGACCAGTCGAGTTACGGTTCCATCCATCCGAACAGCTATGTCAAAACTGCTTAAGCCAAACCCTGAATTG<br>GCTGCTATGATTCATGAAAAGTGTTCAGGGTTAAGCAATTGGTATGGATTAATTCCTGAGCTATTTCCCAAGGCTAAGTACATATATGGTATCATGACAGGTTCGATGGAACCTTATTT<br>GAAAAAATTGAGGCACTACGCAGCGGAGCTACCGTTGCTGAGTGCTGATTATGGGTCTTCTGAAGGGTGGATTGGGGCAAATGTTAATCCAAATTTCCCCCAGAGTCAGCAACTT<br>TTGCCGTGCTTCCCAATATTGGGTATTTCGAATTTATACCCCTGAGAGAGAATCTTGATGGCCTGGCTCAAGATAATGATTCCTCCTTAATCTTCTTAGAGCCTAAGCCAGTGGATATG<br>ACTGAGGTCAAGATTGGCGAGGAATACGAGATCATCATAACCAATTTCGCAGGGTTGTACAGGTATAGATTAGGAGATGTGGTTAAGGTAATGGGATTTCACAACTCGACCCCGGA<br>ACTCCAATTTGTTTGCAGGAGAAACCTTCTACTCAACATCAATATCGATAAGAACACCGAAAAAGATTTACAGCTATCCGTGGAAGCAGCAACCAAAGTGCTAGCTGCAGAAAAA<br>CTCGAGGTTGTGGATTTTACAAGCCGTGTTGATTTGTCCACAGATCCCGGTCATTATGTCATCTTTTGGGAAATAAGTGGCGAAGCAAATGACGAGGTTTTGAAGGAATGCTGCAAT<br>TGTTTGGACAAATCATTTGTGGACGCAGGCTATATGAGCTCCCGCAAAGTCAATGCCATTGGAGCGCTGGAGCTCCGAGTCTTGAGGAGGGGAACCTTTCATGAGATTCTTGATCA<br>TTTTGTAGGATTGGGAGGTGCAGTTAGCCAATTCAAAACTCCGAGGTGCGTCGGACCCAATAATAACACGGTGCTGCAGATTCTATGTAACAATGTTGTTAAGAACTACTTTAGCA<br>CTGCCTATTGA
4	PgJAR4 (PgGH3.7)	ATGTTGGAAAAGATGGAGAAAATATTTGACCATGAAGAATTTATTGAAGAATTTGAGGCTCTGACGAAGGATGCAGGGAGAGTTCAAAAGGAGACCTTAAGGAAAATATTGGAA<br>GAAAATCGAAATACAGAATACTTACAGAAATGGGATCTTGATGGAAAAACTGATCGGGAGAGTTTTGCAAAATGTGTGCCTCTTGTCACTCACAAGGATTTGGAACCTTACATTCA<br>GAGAATCGTGGATGGTGATGTTTCCCCAATTCTCACTGGAAAACCGATAAAAACCATCTCACTAAGTTCCGGCACTACTCAGGGTAAGCCCAAGTTTGTACCTTTCAATGATGATTT<br>GGTGGAATCTACTATGCAGATATATAAGACATCTTTTGCCTTTAGAAACAGAGATTTTCCAATTCGAAATGGAAAGGCCTTGCAGTTTATCTATGGTAGCAAGCAGTTTAGAACCAA<br>AGGGGGACTGCCAGCAGGAACTGCTACTACAAATGTGTATCGTAGTTCACAGTACAAGAAAACAATGAAGGCAATGCAGACCCCGTGTTGTAGCCCGGATGAAGTGATATTTGGT<br>CCTGACTTCCACCAATCCTTGTACTGCCATCTCCTATGTGGACTTATTTTCCGGGATGACGTTCAAGTTGTGTCCTCTACATTTGCTCACAGCATTGTTCATGGCTTCAGGACTTTTGA<br>ACAAGTTTGGGAAGAACTCTGTACTGATATCCAAGAAGGAGTCCTGACCAATCGAGTTACGGTTCCATCCATCCGAACAGCTATGTCAAAACTGCTTAAGCCAGACCCTGAATTG<br>GCTGCTATGATTCATGAAAAGTGTTCAGGGTTAAGCAATTGGTATGGATTAATTCCTGAGCTATTTCCCAAGGCTAAGTACATATATGGTATCATGACAGGCTCGATGGAACCTTATT<br>TGAAAAAATTGAGGCACTACGCAGCGGAGCTACCGTTACTGAGTGCTGATTATGGGTCTTCTGAAGGGTGGATTGGGGCAAATGTTAATCCAAATTTCCCCCAGAGTCGGCAACT<br>TTTGCCGTGCTTCCCAATATTGGGTATTTCGAATTTATACCCCTGAGAGAGAATCTTGATGGCCTGGCTCAAGATAATGATTCCTCCTTAATCTTCTTAGAGCCTAAGCCAGTGGATAT<br>GACTGAGGTCAAGATTGGCGAAGAATACGAGATCATCATAACCAATTTCGCAGGGCTGTACAGGTATAGATTAGGAGATGTGGTTAAGGTAATGGGATTTCACAACTCGACCCCG<br>GAACTCCAATTTGTTTGCAGGAGAAACCTTCTGCTCAACATCAATATCGATAAGAACTCCGAAAAAGATTTACAGCTATCCGTGGAAGCAGCAACCAAAGTTCTAGCTGCAGAAA<br>AACTCGAGGTTGTGGATTTCACAAGCCGTGTTGATTTGTCCACAGATCCCGGTCATTATGTCATCTTTTGGGAAATAAGTGGCGAAGCAAATGACGAGGTTTTGACGGAATGCTGC<br>AATTGTTTGGACAAATCATTTGTGGACGCAGGCTATATGAGCTCCCGCAAAGTCAATGCCATTGGAGCGCTGGAGCTCCGAGTCTTGAGGAGGGGAACCTTTCATGAGATTCTTGA<br>TCATTTTGTAGGATTGGGAGGTGCAGTTAGCCAATTCAAAACTCCGCGGTGCGTCGGACCCAATAATAACACGGTGCTGCAGATTCTATGTAACAATGTTGTTAAGAACTACTTTAG<br>CACTGCCTATTGA
5	Pg_S1240.25 （PgGH3.5）	ATGGTGGTAGACTCAATATTGTTATCACCACTCGGACCTCCGGCCTGCGAAAAAGACGCCAAGGCGCTCCAATTCATAGAAGATATGACTAGAAATGCTGATTTTGTGCAAGAAA<br>AAGTATTAGCTGAAATACTGAGTCGAAACGCTAAGACTGAGTATCTTCAGCGATTCAAACTCCACGGTGCAACAGATCGCGATATATTCAAATCCAAAATCCCCATGGTTACTTAC<br>GAAGATCTTCAGCCTGATATTCAACGTATCGCTAATGGCGATCGCTCTCCAATCTTATCATCTCATCCCATTTCTGAATTCCTCACCAGTTCTGGGACTTCAGCTGGAGAGAGAAAAC<br>TCATGCCCACAATTAAGGAAGAGTTGGATCGTCGCCAACAACTTTACAGTCTTCTCATGCCTGTTATGAACCTTTGCGTGCCTGGTTTGGACAAAGGAAAAGGACTCTACTTTTTGT<br>TTGTGAAGTCAGCAACAAAGACTCCGGGTGGTTTATTGGCCCGCCCGGTACTCACTAGCTACTACAATAGTGATCATTTCAAGACCCGACCCTACGATCCGTACAATGTCATGACA<br>AGCCCCGACGAGGCAATCCTCTGTGTTGATTCTTTCCAAAGCATGTACTCCCAGATGCTATGCGGTCTCTACCACCGCCAAAGCGTTCTCCGGCTTGGGGCCGTGTTCGCCTCCGGC<br>CTTCTCCGGGCCATTCGGTTCCTCCAACTCAATTGGCAAGAACTCGCCCATGATATCCGAACCGGAAGCCTAAACACAAAGGTAACCGACCCGGGTATTCTTTCATGCATGAACCG<br>GGTGATCAGATCGGACCCGGATCTTGCGGAGTTCATATGCAAGGAATGTTCTCATGATAATTGGGAAGGAATTGTCACCAAGATTTGGCCTAATACAAAGTATCTCGACGTAATCGT<br>AACCGGGGCAATGGCTCAATACATCCCAACTCTCAATTTTTATAGTGGCGGATTACCCATGGCATGCACCATGTACGCGTCTTCGGAGTGTTATTTCGGGTTAAACCTCAACCCGAT<br>GTGCAAACCGTCGGAAGTTTCATATACCATCATGCCAAACATGGCCTACTTCGAGTTCCTACCCCACGAGCCCAACTCAACCGGGTCAACTCGCAACTCACCTCCAAAACTCGTT<br>GACCTTGTGGATGTTGAACTAGGCAAAGAATATGAACTTGTAATCACTACCTATGCAGGACTCTATAGGTACCGAGTTGGTGACATACTCAGAGTCACCGGGTTCCACAACTCAGC<br>ACCGCAATTCCACTTCGTGAGGCGAAAAAACGCACTACTCAGCATTGACTCAGACAAGACCGACGAGGCCGAGCTACAGACGGCAGTTGAGAACGCGTCCCAACTCCTCCCCG<br>AATTCAACACGAGCGTAGTCGAGTACACGAGTTACGCAGACACGAAGACGATCCCGGGTCACTACGTGATATATTGGGAGTTATTAGTGAAAGACTCGGTGAACTCGCCTAGTGA<br>TGAGGTGCTAAATCAGTGTTGCCTAGCTATAGAAGAGTGTCTTAACTCAGTGTATAGACAATGCCGAGTTGCGGACAACTCGGTGGGGCCACTTGAAATACGTGTAGTGAAGAATG<br>GAACATTTGAAGAGCTAATGGATTATGCAATATCAAGAGGGGCGTCCATTAATCAATATAAGGCGCCGAGGTGTGTTAACTTTACACCAATTATGGAGCTGCTCGACTCCAGAGTG<br>GTTTCATCGCACTTTAGTCATAGTTTGCCTCATTGGACCCCAGAACGACGCCGGTAA
6	Pg_S1464.2 （PgGH3.4）	ATGGCGGTTGACTCTACAATCTTCTCTCCTCTTGGGCCTCCTGCCTGCGAGAAAGACGCCAAGCCTCTTCAGTTCATTGAAGAGATGACGAAAAATCCAGACTCGGTTCAAGAGA<br>ACGTTTTGACCGAAATATTGAGCCGAAATGCCGAGACAGAGTACCTGAAAACGTTCCACCTCGATGGTGCCACGGACCGGGACACTTACAAGTCAAAGGTGCCGGTTGTTAGTTA<br>TGAGGACCTTCAGCCCTATATTCAACGGATCGCTAATGGTGACCGTTCTCCCATACTGTCTTCCCATCCCATCTCCGAGTTTCTCACTAGTTCTGGGACATCTGCTGGGGAAAGGAA<br>GCTCATGCCAACGATTGCGGAAGAATGGACCGTAGACAGAAGCTATACAGCCTTCTCATGCCTGTCATGAATCTGTATGTGCCAAATTTGGACAAGGGGAAGGGTTTGTACTTTC<br>TGTTTATAAAGGGGGAAACGAAGACCCCGAGCGGGTTAGTAGCACGTCCCGTGCTGACCAGTTATTACAAGAGTGAACAATTCATGAGCAGGACATATGACCCTTACAATGTTTA<br>CACCAGTCCTGACGAGGCCATTCTCTGTGCCGATTCTTTCCAAAGCATGTACTCTCAGATGCTATGTGGCCTCCTTATGCGCCAACAAGTCCTTCGAATCGGCGCCGTCTTCGCCTC<br>CGGCCTAGTTCGAGCCATTCGCTTCCTCCAACTCAATTGGCCACAACTGGCCCATGACATTGGTTCCGGGACTTTGAATCCTAAAGTTACAGACCCGTCCATCAGAGCACGCATGT<br>CCGATATACTCAAACCCGACCCGGAATTGGCCGAGTTCATTACTCAACAGTGCTCGGATGAGAACTGGGAAGGGATTATCACAAGAATTTGGCCAAATACTAAGTATCTGGACGT<br>GATTGTTACGGGTGCGATGGCGCAGTACATTCCAACACTTGATTATTATAGTGACGAGTTGCCAAAATATTGCACCATGTATGCATCCTCTGAGTGCTATTTTGGGCTTAATTTAAAG<br>CCAGTGTGCAAGCCATCTGAAGTTTCTTACACCATCATGCCCAACATGGGATACTTTGAGTTCCTCCCCCACGGCCCGTCCTCTCCAGTGACTCTCTCACGTGACTCGCCGCCCCGC<br>CTCGTCGACCTCGCCGACCTAGAAGTGGGGAAGGAATATGAACTTGTGATTTCGACCTATGCCGGGCTGTGTCGTTACCGAGTTGGCGATATTCTACGAGTCACCGGGTTCTATAAC<br>TCGGCGCCTCAGTTCAAGTTTATAAGGAGGAAAAACGTTTTGCTAAGCATCGACGCGGACAAAACGGACGAGGCGGAATTACAAAGAGCCACTGATAATGCCTCGGTTTTGTTAC<br>GTGAATTTAATACTAGCATAGAGGAATACACTAGCTATGGTGACACGAAAACCATACCGGGGCATTATGTTATTTATTGGGAATTGCTAGTGAAAGATCCGTCGAACCCACCGAGTC<br>ACGAGGTGTTGAACCGGTGTTGTTTAGCAATGGAGGAATCGTTGAACACGGTGTACCGACAAGGTCGAGTCGCGGACAACTCGATCGGACCGCTTGAAATAAGGGTGGTGAAAA<br>ATGGGACATTTGAGGAGCTTATGGATTATGCAATTTCTAGGGGTGCGTCAATTAATCAATATAAGGTGCCGAGGTGTGTTAGTTTCGCACCCATAAAGGAACTACTTGACTCGAGAG<br>TGGTCTCGGCGCACTTCAGCCCAGCTGCCCCGCATTGGTCCTCAGAACGACGACGCTGA

7	Pg_S2160.32 (PgGH3.19)	ATGGCGGTTGACTCAACCATCTCGTCTCCTCTCGGCCCTCCGGCATGCGAGAAAGATGCCAAGGCTCTTCAATTTATCGAAGAAATGACCAAAAATGCAGATTCAGTACAAGAGA<br>AAGTTTTGGCCGAAATATTGAGCCGAAATGCTGAAACTGAATACCTCCGAAACTTCAACCTCAACGGTGCCACGGACCGCGACACCTATAAGTCCAAAGTGCCGGTTGTTAATTA<br>CGAGGACCTCCAGCCCTATATCCAACGGATCGCTAATGGCGACCGCTCTCCCATCCTTTCTTCTCACCCCATCTCGGAGTTTCTTACTAGTTCAGGAACATCTGCTGGAGAGAGAAA<br>GTTGATGCCAACCATCGCTGAAGAGTGGGACCGTAGGCAGAAGCTATACAGCCTTCTCATGCCAGTCATGAATCTCTACGTGCCGAATTTGGACAAAGGAAAGGGTTTGTACTTTC<br>TGTTTATCAAGGCGGAAACCAAGACCCTGAGCGGATTAGTAGCACGTCCGGTGCTGACCAGTTACTACAAGAGTGAACAGTTCATGACCCGTCCATATGACCCATACAATGTCTAC<br>ACTAGCCCTGATCAGACCATTCTTTGTGCTGACTCTTTCCAAAGCATGTATTCTCAAATGCTTTGTGGCCTCGTCATGCGCCAACAAGTCCTTCGAATGGGTGCCGTTTTTGCCTCCG<br>GCCTAGTCCGAGCCATCCGCTTCCTCCAGCTCAATTGGAGACAACTGGCCGATGACATTGCATCCGGGGTTTTGAACCCAAAAATATCAGACTCGTCTATCAGACTATGCATGTCG<br>GGTATTTTAAAACCCGACCCGGAGCTTGCTGAATTTATCACTCAGCAGTGCTCGGACGAGAATTGGGAGGGGATTATTACTAGAATTTGGCCAAATACCAAGTATTTGGACGTGATT<br>GTTACGGGTGCAATGGCTCAGTACATTCCAACGCTTGATTATTATAGCAATGAGTTGCCTAACTATTGCACCATGTATGCGTCTTCCGAGTGCTATTTTGGGCTTAACCTAAAGCCCG<br>TTTGCAAGCCATCTGAAGTTTCTTATACCATCATGCCAAATATGGGCTACTTTGAATTTCTCCCCCACGATTCGTCGGCTCCGGTGACTCTCTCCTGTGACTCGCCACCTCGGCTTGTT<br>GACCTTGCTGAGCTAGAAGTGGGGAAGGAGTACGAACTCACCATTTCGACCTATGCGGGACTGTGTCGATACCGAGTTGGTGACATCCTCCAAGTCACCGGGTTCCATAACTCGG<br>CCCCGCAGTTCAAGTTCATAAGGAGGAAGAACGTTTTGCTAAGTATTGACTCGGACAAGACCAATGAGGCCGAGTTACAAAGCGCCATTGATAATGCGTCGGTGTTGTTACGTGA<br>ATTTAATACAACTATAGTGGAATACACTAGCTATGGTGACACCAATACCATCCCGGGGCATTATGTTATTTATTGGGAATTACTAGTGAAAGACGCGGCCAACTCACCGAGTCATGA<br>CGTGTTGAACCAATGTTGTCTAGCAATGGAAGAATCGTTGAACTCGGTGTACCGACAAGGCCGCGTGGCGGACAACTCAATCGGACCACTTGAAATAAGAGTGGTGAAAAACGG<br>CACATTTGAGGAGCTGATGGATTATGCAATTTCTAGGGGTGCGTCAATTAATCAATACAAGGTTCCAAGATGCGTAAGTTTCACACCCATTATGGAACTCCTTGACTCGAGAGTTGT<br>GTCGGCGCATTTTAGCCCCGCGTCCCCACAATGGACCCCAGAACGACGACGTTGA
8	Pg_S2668.23 (PgGH3.21)	ATGGCGGTTGACTCTACAATCTCCTCTCCTCTTGGGCCTCCTGCCTGCGAGAAAGACGCCAAGGCTCTTCAATTCATTGAAGAGATGACGAAAAATCCAGACTCGGTTCAAGAGA<br>ACGTTTTGGCCAAAATATTGAGCCAAAATGCCGAGACAGAGTACCTGAAAACGTTCCACCTCGATGGTGCCACGGACCGGGACACTTACAAGTCAAAGCTGCCGGTTGTTAGTTA<br>TGAGGACCTTCAGCTCTATATTCAACGGATCGCTAATGGTGACCGTTCTCCCATACTGTCTTCCCATCCCATCTCCGAGTTTCTCACTAGTTCTGGGACATCTGCTGGGGAAAGGAA<br>GCTCATGCCAACGATTGCGGAAGAATGGGACCGTAGACAGAAGTTATACAGCCTTCTCATGCCTGTCATGAATCTGTATGTGCCAAATTTGGACAAGGGGAAGGGTTTGTACTTTC<br>TGTTTATAAAGGCGGAAACGAAGACCCCGAGCGGGTTAGTAGCACGTCCCGTGCTGACCAGTTATTACAAGAGTGAACAATTCATGAGCAGGACATATGACCCTTACAATGTTTA<br>CACCAGTCCTGACGAGGCCATTCTTTGTGTCGATTCTTTCCAAAGCATGTACTCTCAGATGCTATGTGGCCTCCTTATGCGCCAACAAGTCCTTCGAATGGGCGCCGTCTTCGCCTCC<br>GGCCTAGTTCGAGCCATTAGCTTCCTCCAACTCAATTGGCCTCAACTGGCCCATGACATTGGTTCCGGGACTTTGAACCCTAAAGTTACAGACCCGTCCATCAGAGCACGCATGTC<br>CGATATACTCAAACCCGACCCGGAATTGGCCGAGTTCATCACTCAAGAGTGCTCGGATGAGAACTGGGAAGGGATTATTACAAGAATTTGGCCAAATACTAAGTATCTGGACGTG<br>ATTGTTACGGGTGCGATGGCCCAGTACATTCCAACACTTGATTATTATAGTAACGAGTTGCCAAAATATTGCACCATGTATGCATCCTCTGAGTGCTATTTTGGGCTTAATTTAAAGCC<br>AGTGTGCAAGCCATCTGAAGTTCGTTACACCATCATGCCCAACATGGGCTACTTTGAGTTCCTCCCCCACGGCCCGTCCTCTCCAGTGACTCTCTCACGTGACTCGCCGCCCCGCCT<br>CGTCGACCTCGCCGACCTAGAAGTGGGGAAGGAATATGAACTTGTGATTTCGACCTATGCCGGGCTGTGTCGTTACCGAGTTGGCGATATTCTACGAGTCACCGACTTCTATAACTC<br>GGCGCCTCAGTTCAAGTTCATAAGGAGGAAAAACGTTTTGCTAAGCATCGACGCGGACAAAACGGACGAGGCGGAATTACAAAGTGCCATTGATAATGCCTCGGTTTTGTTACGT<br>GAATTTAATACTAGCATAGTGGAATACACTAGCTATGGTGACACGAAAACCATACCGGGGCATTATGTTATTTATTGGGAATTGCTAGTGAAAGATCCGTCGAACCCACCGAGTCAC<br>GAGGTGTTGAACCGGTGTTGTTTAGCAATGGAGGAATCGTTGAACACGGTGTACCGACAAGGTCGAGTCGCGGACAACTCGGTCGGACCGCTTGAAATAAGGGTTGTGAAAAAT<br>GGGACATTTGAGGAGCTTATGGATTATGCAATTTCTAGGGGTGCGTCAATTAATCAATATAAGGTGCCGAGGTGTGTTAGTTTCGCACCCATAATGGAACTACTTGACTCGAGAGTG<br>GTCTCGGCGCACTTCAGCCCAGCTGCCCCGCATTGGTCCTCAGAACGACGACGTTGA
9	Pg_S3044.20 (PgGH3.17)	ATGGCGGTAGACTCAATATTGTCATCACCACTCGGACCTCGGGCCTGCGAAAAAGACGCCAAGGCGCTCCAATTCATAGAAGAGATGACTAGAAATGCTGATTTTGTGCAAGAAA<br>AAGTATTAGCTGAAATACTGAGTCGAAACGCTAAGACTGAGTATCTTCAGCGATTCAAACTCCACGGTGCAACAAATCGCGATATATTCAAATCCGAAATCCCCATGGTTACTTAC<br>GAAGATCTTCAGCCCGATATTCAACGTATCGCTAATGGCGATCGCTCTCCAATCTTATCATCTCATCCCATTTCCGAATTCCTCACCAGTTCTGGGACTTCAGCTGGAGAGAGAAAA<br>CTCATGCCCACAATTAAGGAAGAGTTGGATCGTCGCCAACAACTTTACAGTCTTCTCATGCCTGTTATGAACCTTTGCGTGCCTGGTTTGGACAAAGGAAAAGGACTCTACTTTTTG<br>TTTGTGAAGTCAGCAACAAAGACTCCGGGTGGTTTATTGGCCCGCCCGGTACTCACTAGCTACTACAATAGTGATCATTTCAAGACCCGACCCTACGACCCGTATAATGTCATGAC<br>AAGCCCCGACGAGGCAATCCTCTGTGTTGATTCTTTCCAAAGCATGTACTCCCAGATGCTATGCGGTCTCTACCACCGCCAAAGCGTTCTCCGGCTTGGGGCCGTGTTCGCCTCTGG<br>CCTTCTCCGGGCCATTCGGTTCCTCCAACTCAATTGGCAAGAACTCGCCCATGATATCCGAACCGGAAGCCTAAACACAAAGGTAACCGACCCGGGTATCCGTTCATGCATGAAC<br>CGGGTTATCAGATCCGACCCGGATCTTGCGGAGTTCATATGCAAGGAATGTTCTCATGATAATTGGGAAGGAATTGTCACCAAGATTTGGCCTAATACAAAGTATCTCGACGTAATA<br>GTAACCGGGGCAATGGCTCAATACATCCCAACTCTCAATTTTTATAGTGGCGGATTACCCATGGCATGCACCATGTACGCGTCTTCGGAGTGTTATTTCGGGTTAAACCTCAACCCG<br>ATGTGCAAACCGTCGGAAGTTTCATATACCATCATGCCAAACATGGCCTACTTCGAGTTCCTACCCCACGAGCCCAACTCAACCGGGTCAACTCGCAACTCACCTCCAAAACTCGT<br>TGACCTTGTGGATGTTGAACTAGGCAAAGAATATGAACTTGTAATCACTACCTATGCAGGACTCTATAGGTACCGAGTTGGTGACATACTCAGAGTCACCGGCTTCCACAACTCAG<br>CACCGCAATTCCACTTCGTAAGGCGAAAAAACGCACTACTCAGCATTGACTCAGACAAGACCGACGAGGCCGAGCTACAGACGGCAGTTGAGAACGCGTCCCAACTCCTCCGC<br>GAATTCAACACGAGCGTAGCCGAGTACACGAGTTACGCAGACACGAAGACGATCCCGGGTCACTACGTGATATATTGGGAGTTATTAGTGAAAGACTCGGGGAACTCGCCTAGTG<br>ATGAGGTGCTAAATCAGTGTTGCCTAGCTATGGAAGAATGTCTTAACTCAGTGTATAGACAATGCCGAGTTGCGGACAACTCGGTGGGGCCACTTGAAATACGTGTAGTGAAGAAT<br>GGAACATTTGAAGAGCTAATGGATTATGCAATATCAAGAGGGGCGTCCATTAATCAATATAAGGCGCCGAGGTGTGTTAACTTTACACCAATTATGGAGCTGCTCGACTCCAGAGT<br>GGTTTCATCGCATTTTAGTCATAGTTTGCCTCATTGGACCCCAGAACGACGCCGGTAA
10	Pg_S8450.1 (PgGH3.1)	ATGGCGGTTGACTCAACCATCTCGTCTCCTCTCGGCCCTCCGGCATGCGAGAAAGATGCCAAGGCTCTTCAATTTATCGAAGAAATGACCAAAAATGCAGATTCAGTACAAGAGA<br>AAGTTTTGGCCGAAATATTGAGCCGAAATGCTGAGACTGAATACCTCCGAAACTTCAACCTCAACGGTGCCACGGACCGCGACACCTATAAGTCCAAAGTGCCGGTTGTTAATTA<br>CGAGGACCTCCAGCCCTATATCCAAAGGATCGCTAATGGCGACCGCTCTCCCATCCTTTCTTCTCACCCCATCTCCGAGTTTCTTACTAGTTCAGGAACATCTGCTGGAGAGAGAAA<br>GTTGATGCCAACCATCGCTGAAGAGTGGGACCGTAGGCAGAAGCTATACAGCCTTCTCATGCCAGTCATGAATCTCTACGTGCCGAATTTGGACAAAGGAAAGGGTTTGTACTTTC<br>TGTTTATAAAGGCGGAAACCAAGACCCTGAGCGGATTAGTAGCACGTCCGGTGCTGACCAGTTACTACAAGAGTGAACAGTTCATGACCCGTCCATATGACCCATACAATGTCTAC<br>ACTAGCCCTGATCAGACCATTCTTTGTGCTGACTCTTTCCAAAGCATGTATTCTCAAATGCTCTGTGGCCTCGTCATGCGCCAACAAGTCCTCCGAATGGGTGCCGTTTTTGCCTCCG<br>GCCTAGTCCGAGCCATCCGCTTCCTCCAGCTCAATTGGAGACAACTGGCCGATGACATTGCATCCGGGGTTTTGAACCCTAAAATATCAGACCCGTCTATCAGACTATGCATGTCG<br>GGTATTTTAAATCCCGACCCGGAGCTTGCTGAATTTATCACTCAGCAGTGCTCGGAGGAGAATTGGGAGGGGATTATTACTAGAATTTGGCCAAATACCAAGTATTTGGACGTGATT<br>GTTACGGGTGCAATGGCTCAGTACATTCCAACGCTTGATTATTATAGCAATGAGTTGCCTAAATATTGCACCATGTATGCGTCTTCCGAGTGCTATTTTGGGCTTAACCTAAAGCCCG<br>TTTGCAAGCCATCTGAAGTTTCTTATACCATCATGCCAAATATGGGCTACTTTGAATTTCTCCCCCACGATTCGTCGGCTCCGGTGACTCTCTCCTGTGACTCGCCACCTCGGCTTGTT<br>GACCTTGCTGAGCTAGAAGTGGGGAAGGAGTACGAACTCATCATTTCGACCTATGCGGGACTGTGTCGATACCGAGTTGGTGACATCCTCCAAGTCACCGGGTTCCATAACTCGGC<br>CCCGCAGTTCAAGTTCATAAGGAGGAAGAACGTTTTGCTAAGTATTGACTCGGACAAGACCAATGAGGCCGAGTTACAAAGCGCCATTGATAATGCCTCGGTGTTGTTACGTGAAT<br>TTAATACAACTATAGTGGAATACAGTAGCTATGGTGACACCAAGACCATCCCGGGGCATTATGTTATTTATTGGGAATTACTAGTGAAAGACGCGGCCAACTCACCGAGTCATGAC<br>GTGTTGAACCAATGTTGTCTAGCAATGGAAGAATCGTTGAACTCGGTGTACCGACAAGGCCGCGTGGCGGACAACTCAATCGGACCACTTGAAATAAGAGTGGTGAAAAACGGC<br>ACATTTGAGGAGCTGATGGATTATGCAATTTCTAGGGGTGCGTCAATTAATCAATATAAGGTTCCAAGATGCGTAAGTTTCACACCCATTATGGAACTCCTTGACTCGAGAGTTGTG<br>TCGGCGCATTTTAGCACCGCGGCCCCACAATGGACACCAGAACGACGACGTTGA
11	Pg_S0258.5 (PgGH3.15)	ATGTTGCAGAGACCAAAGGAAATGGCTGCCACTGCATACCCTTTGCACGGAAACAAATCGAATTTGTCCCACTTCCTCTCTATGTTTGTTAGTCCCAAAGAAGTAATTCTAGGATC<br>GGATGTTCAGCAGCAGATGTATTGTCACCTCCTTTGTGGTCTTAGGCTTTCCGATTTTGTTGATTCAATTCTAGCACCATATGCTGAGGGTTTTATTATAGCATTTAGGCTTCTAGAGTC<br>TAATTGGGAGAAGCTATGCGAGGATCTTGAGAATGGGTTTCCGAGTTTGGATGTTACTGATGTTGCAATGAGAGATTCAGTTGCTGAGGTTCTTTCTGGTCCCCAAGTAGATCTGTC<br>GAAGAGGATTAGATCAATATGTGAAGAACTGAATTGGGAATGGGTTGTTACTAAGCTTTGGCCAAATGTTCGATATGTTAAGTGTGTTACTACCGGAAGTATGGAGCTATATTATTCA<br>AATCTCAAATACTAA
12	Pg_S1142.14 (PgGH3.10)	ATGTCTGCCGTGGCTAGTTTTCAACTTATCTTAAGAGAAGTGATGTCGGCAGAGATTGTGGAGTTCACAGGTTTTTTGGACCTTGAATTGAATCCTCGGCAATTGAAGTGCAAAAAA<br>AACTGTTTCCCAAGTGATCAAGTCAAGAATCATTTGAACCTCAGATGTAGGGTGTGTTTTCAGATACAAGAAGGTTCTTCTGACGTTCAAGAATTTCTTGAGGAATTCCTTAGCATG<br>TGGAGATGTGTGGATGACAAGTATTATGTTCTTAAAAGAGCAGAAGAAAATATAAGTAATTTGGGAGGATGTGACAACCCATTTGTGCTAGGAGTTGATAAGTACATTGAAGTTGT<br>TGAGGTTTATGTTGTAACACTTATTGCAATGCTTTTGAGGGACATGGATCTTGCCATCTCTTGGGTTGAGAAAGCTGCATTACCTGAGCAAAAGAGACAGAGACCAAAGGAAATGG<br>CTGCCACTGCATACCCTTTGCACGGAAACAAATCGAATTTGTCCCACTTCCTCTCTATGTTTGTTAGTCCCAAAGAAGTAATTCTAGGATCGGATGTTCAGCAGCAGATGTATTGTCA<br>CCTCCTTTGTGGTCTTAGGCTTTCCGATTTTGTTGATTCAATTCGAGCACCATATGCTGAGGGTTTTATTATAGCATTTAGGCTTCTAGAGTCTAATTGGGAGAAGCTATGCGAGGATC<br>TTGAGAATGGGTTTCCGAGTTTGGATGTTACTGATGTTGCAATGAGAGATTCAGTTTCTGAGGTTCTTTCTGGTCCCCAAGTAGATCTGTCAAAGAGGATTAGATCAATATGTGAAG<br>AACTGAATTGGGAATGGCTTGTTACTAAGCTTTTGCCAAATGTTCGATATGTTAAGTGTGTTACTACCGGAAGTATGGTGCAATATTATTCAAATCTCAAATACTACGCAGGAGAGAT<br>ACCACTATTAGGTGGAGGCTACTTTACCTCAGAGTGCTCTGTGGGAATTAACTTTAATATTATGCAACCTCCAGAACTGACTCGCTTTGTTATACTTCCAACTGCAGCATATTTTGAAT<br>TTCTTCCGTTTGACTGGAACAGGGCCCCTGTCACGGATGAACAAACAGTTGATCTTTCTGGTGTTGAGCTGGGGATGAAATGTATGAGGTGGTTGTTTGGGTGA
13	Pg_S2504.6 (PgGH3.12)	ATGATGTCGGACGACGATATCATCGCAAAGCTCGAGGACACCACTAAAGACGCCACGCGCCACCAGCTGGAGACACTCCAAACCATCCTCGACCGGAACGCCGGCGCAAGTTAT<br>CTCCAGCCCTACCTCCGAAGTTATCATGCGCCCGTCGACGCCTCTACTTTCCGGCGAGCCGTGCCGTTATCTTGCTACGATGACTACGCTGATCACATACATCAACTCGCCGACGGC<br>GTTCTTTTCGACGATGATGCTCGGCCATTACTGACCCTTGATCCCCTGCTATGTTTCTTTTACAGTTCAGGGACAGGTTCCAAGAAGCCGAAATTGATTCCTTATTTTGATTCACCATC<br>TTCAAAGGCCATCTCCTTTTTAGCTCATCAAGGTAGCCTAGCCATTCTTCGTAGGTTGTTTCCACCAAAACTCTCGATCAATAAGAGCCTATGGTTTTACTATGCGGGAAATGTTGCA<br>GAGACCAAAGGTGGATACAAGGCTATGGCTGCCACTGCATACGCTATGCACGGAAACAAATCAAATTTGTCCCACTTCCTCTCTATGTTTGTTAGTCCCAAAGAAGTCATTCTAGG<br>ATCGGATTTTCAGCAGCAGATGTATTGCCACCTCCTTTGTGGTCTTAGGCTTTTCGATTTTGTTGATTCAATTCGAGCACCATATGCTGCAGGTTTGATTATAGCATTTAGGCTTCTAGA<br>GTCTAATTGGGAGAAGCTATGCGAGGATCTTGAGAATGGATTTCCGAGTTTGGATGTTACTGATGTTGCAATGAGAGATTCAGTTACTGAGGTTCTTTCTGGTCCCCAAGTAGATCT<br>GTCGAAGAGGATTAGATCAATATGTGAAGAACTGAATTGGGAAGGGATTGTTACTAAGCTTTGGCCAAATGTTCGATATGTTAAGTGTGTTACTACCGGAAGTATGGAGCAATATTA<br>TTCAAAACTCAAATACTATGCAGGAGAGATACCACTGTTAGGTGGAGACTACTTTGCCTCAGAGTGCTCTGTGGGAATTAACTTTAATATTATGCAACCTCCAGAACTGACCCGCTT<br>TGTTATACTTCCAACTGCAGCATATTTTGAATTTCTTCCGTTTGACTGGAACAGGGCCCTTGTCACGGATGAACAAACAGTTGATCTTTTTGGTGTTGAACTGGGGAAAATGTATGA<br>GGTGGTTGTGACTACTTATAGAGGATTGTACCGGTACTGTTTGGGTGACATTGTGAGGGTTGTTGGCTTTTATAACTCATCTCCTGAGGTTGAGTTTGTGATGAGAGCTCCTAACGCT<br>TCTAGTGATATAATAACTGAAGGAGATCTCATGTCTGCCATGGGTAGTTTTCAACTTATCTTAAGAGAAGTAATCTCAGCAGAGATTGTGGAGTTCACAGGTTTTTTGGACCTTGAAT<br>TGAATCCTCGGCAATTGAAGGTATTTATTGAAGTTAAAGAAGGATGTATATTTCTGCAGGAGGAGAAGTTGAAGGAGCTGATAATAATTCTTAAAAACTGTTCCTTCTCTCTTGAGG<br>ATGGTTTAGGAGGTATTTATAAAGTGATAAAGGCTAGAGGTGAAATTGGTCCTTTGATAGTATCCATTGTAAGGCCAGGCAGTTTTGATTGCTTATCAGAAAAGGCCATAGAAAGTG<br>GGGCACCGGCAAGTCAATATAAACCCCCAAAAATTATAAGTAATCGCAAATTTGCTGATTTACTGGAAATGTCCGTTGTTGTGACTGTTTGCTTGAATTCTTTGAATGGTTAA

14	Pg_S4448.3 (PgGH3.8)	ATGCCTGAAGCTCCAAAAAATTACCCCCACAAGCTCACAGAATTAAGTGTCCTTACAGAGAAGAACAAAAAGGCTCTTCAGTTCATTGAAGATGTCACCTCTAACGCCGATGAGGTTCAGAAGCGGGTTCTTGCCGAGATTCTCTCCCGCAGTTCCCATGTAGAATACCTGCGGCGATACGGCCTCGACGGCCACACCAACAGGGACACTTTCAAAAAAATCATGCCCGTCATCAAATATGAGGATTTACAGCCTGATATCAACCGTATTGCCAATGGTGATAACTCCCCAATCCTTTGCTCTGACCCCATTTCCGAGTTCTTAACAAGCTCTGGGACATCTGGAGGAGAGAGAAAACTAATGCCCACCATTGAAGAAGAGCTAGAGAGGAGATCGCTGCTGTATAGCCTTTTGATGCCTGTGATGAACCAGTCTGTTCCTGGTCTAGACAAAGGCAAAGGAATGTATTTCTTGTTTATTAAATCTGAGGCTAAGACTCCTGGTGGCCTACTAGCTCGTCCCGTTTTAACTAGTTACTACAAAAGTTCCCATTTCAAAAACAGGCCTTATGACCCGTACACAAACTACACTAGCCCAAATGAGACCATTCTCTGCCCAGACTCCTACCAGAGCATGTACTCCCAAATGCTCTGTGGGCTATGCCTTAACAAGGAAGTCCTTAGGGTCGGAGCTGTTTTTGCCTCCGGCTTCATCCGGGCCATCCGGTTCCTTGAAAAGCACTGGTCCATTCTCTGTAACGACATCCGAACCGGAACTCTTAATCCTGAAATCACCGACCCATCGGTTAGAGAATCGGTGATGAAAATCCTCAAACCCGACACCAAATTAGCGGATTTTATTGAGGCAAAATGTAGTAAAAAGTCATGGCAAGGGATCATAACTAGGCTGTGGCCTAACACAAAGTATGTTGATGTTATTGTGACAGGGACCATGTCACAGTATATACCAACTCTTGATTACTATAGCAATGGACTCCCTCTTGTGTGCACCATGTATGCTTCTTCAGAGTGCTACTTTGGTGTCAATCTTAACCCTCTTTGCAAGCCTAGTGAAGTCTCCTATACCCTCATCCCAACCATGGCCTATTTTGAATTTTTGCCTGTCCATAGAAACAATGGTACTGTCACAAATTCCATAACCATGCCCAAATCCCTCAATGAGAAGGAACAGCAAGAATTGGTTGATCTGGCAGATGTAAAGCTCGGACAAGAATATGAGCTTGTTGTCACCACCTATGCTGGGCTTTATAGATACAGAGTGGGAGATGTGCTTCGAGTTGCTGGATTCAAGAATAAGGCCCCCCAATTCAACTTTATTTGCCGCAAAAATGTGGTTCTAAGCATCGATTCAGACAAAACTGATGAAGTTGAACTTCAAAACGCGCTTAGTGTGAATAGATCAACCCCAATTCCTCCATCTGTTTTTGAGGATTGTTGCCTTGCGATTGAAGAGTCTCTCAACAGTGTGTACCGCCAAGGCCGTGTCTCGGACAAATCCATTGGTCCCCTTGAGATCAAGATTGTGGAATCAGGGACTTTTGATAAGCTTATGGATTATGCCATTAGCTTAGGTGCTTCAATTAACCAGTACAAGACTCCCCGGTGCGTGAAATATGCACCAATTGTTGAGCTTCTGAACTCAAGAATTGGGTCCAACTACTTCAGTCCAAAATGCCCAAAGTGGGTTCCGGGGCATAAGCAATGGAACAACATGAACTGA
15	Pg_S6397.3 (PgGH3.20)	ATGCCCGAGGAATTATCTAGGGAAATGGACCCAGTAGAATATAATGTCGACACGAGAAATAAGAAGGCACTCCAATTCATCGAAGATGTTACTAACAATGCAGAAGAAATTCAGAAGCAAGTGCTTAACGAAATTCTTTCTCGCAATGCTGATGTTGAGTATTTGCAGAGGCATGGCCTTAATGGCCATGTCCACGAAGAAACTTTTAAGGTGGTCATGCCTGTGGTAACATATGAGGATCTTCAGCCAGATATCGACCGAATTTCCAATGGTGATACTTCACCAATTCTTTGTTCACAGCCCATTTCGGAGTTCTTGACAAGCTCCGGAACATCTGGAGGAGAGAGAAAACTGATGCCAACTATTGAAGAGGAGCTTGGGAGGAGATCATCACTCTATAGCCTTTTGATGCCTGTCATGGACCAATTTGTTCCTGGTTTGGACAAGGGCAAAGGAATGTACTTTTTGTTCACAAAATCCGAAGCCAAGACTCCAGGTGGTCTTCTGGCTCGTCCAGTTCTAACCAGTTACTACAAAAGCTCATATTTTACGGACAGACCTAATGATCCATACACAAACTACACAAGCCCTAATGAAACCATTCTCTGCTCCGACTCTTATCAGAGCATGAGGGGGTTGTGGAAAGGGATCATTTCACGATTATGGCCTAACACCAAGTATATAGATGTTATTGTGACTGGAACAATGTCACAATATATTCCAATTATTGATTACTATAGCAATGGCCTCCCATTAGTTTGCACCATGTATGCTTCTTCCGAGTGTTATTTTGGTGTAAACCTCAACCCTCTTTGCAATCCAAGTGATGTCTCTTACACTCTTATTCCCACCATGGCCTATTTCGAGTTCTTGCCGTTCTATAGGAAACATGAAGTCAATGAATCAACAAAGCTCAATCCCAAGGAACAACTTGAGTTGGTTGATCTTGTCGACGTAAAATTGGGATATGAATATGAGCTTGTGGTCACCACTTATGCTGAATACACAAGCTACGCAGACACATCCATAATCCCAGGCCATTATGTGTTATATTGGGAAATTGGATTAAGCAGCAATGATACCACCGTAATCCCTCCTCCTTCAGTTTTTGAGGATTGTTGCCTTACAATTGAAGAATCACTCAACAGTGTGTACCGTCAGGGTCGGGTATCGGATAAGTCAATCGGACCTTTGGAAATAAAGATAGTAGAAAGTGGGACATTTGATAAGCTCATGGACTATGCTGTTAGCAACGGGGCATCAATAAACCAGTACAAGGCTCCCCGGTGCGTGAAATATGCACCCATTATCGACCTGTTGAACTCAAGGGTGGTTTCCAAATACTTCAGCCCCAAATGCCCCAAATGGGTACCGGGTCACAAGCAGTGGTGCACCAATAACTGA
16	Pg_S7369.1 （PgGH3.3）	ATGATGTCGGACGACGATATCATCGCAAAGCTCGAGGACACCACTAAAGACGCCACGCGCCACCAGCTGGAGACACTCCAAACCATCCTCGACCGGAACGCCGGCGCAAGTTATCTCCAGCCCTACCTCCGAAGTTATCATGCGCCCGTCAACGCCTCTACTTTCCGGCGAGCCGTGCCGTTATCTTGCTACGATGACTACGCTGATCACATCAATCAACTCGCCGACGGCGTTCTTTTCGACGATGATGCTCGACCATTACTGACCCTTGATCCCCTGCTATGTTTCTTTTACAGTTCAGGGACAAGTTCCAAGAAGCCGAAATTGATTCCTTATTTTGATTCACCATCTGCAAAGGCCATCTCCTTTTTAGCTCATCAAGGTAGCCTAGCCATTCTTCGTAGGTTGTTTCCACCAAAACTCTCGATCAATAAGAGCCTATGGTTTTACTATGCGGGAAATGTTGCAGAGACCAAAGGTGGATACAAGGCTATGGCTGCCACTGCATACGCTATGCACGGAAACAAATCAAATTTGTCCCACTTCCTCTCTATGTTTGTTAGTCCCAAAGAAGTCATTCTAGGATCGGATTTTCAGCAGCAGATGTATTGCCACCTCCTTTGTGGTCTTAGGCTTTTCAATTTTGTTGATTCAATTCGAGCACCATATGCTGCAGGTTTGATTATAGCATTTAGGCTTCTAGAGTCTAATTGGGAGAAGCTATGCGAGGATCTTGAGAATGGGTTTCCGAGTTTGGATGTTACTGATGTTACAATGAGAGATTCAGTTGCTGAGGTTCTTTCTGGTCCCCAAGTAGATCTGTCGAAGAGGATTAGATCAATATGTGAAGAACTGAATTGGGAAGGGATTGTTACTAAGCTTTGGCCAAATGTTCGATATGTTAAGTGTGTTACTACCGGAAGTATGGAGCAATATTATTCAAAACTCAAATACTATGCAGGAGAGATACCACTGTTAGGTGGAGACTACTTTGCCTCAGAGTGCTCTGTGGGAATTAACTTTAATATTATGCAACCTCCAGAACTGACCCGCTTTGTTATACTTCCAACTGCAGCATATTTTGAATTTCTTCCGTTTGACTGGAACAGGGCCCCTGTCACGGATGAACAAACAGTTGATCTTTTTGGTGTTGAGCTGGGGAAAATGTATGAGGTGGTTGTGACTACTTATAGAGGATTGTACCGGTACTGTTTGGGTGACATTGTGAGGGTTGTTGGCTTTTATAACTCATCTCCTGAGGTTGAGTTTGTGATGAGAGCTCCTAAAGCTTCTAGTGATGTAATAACTGAAGGAGATCTCATGTCTGCCATGGGTAGTTTTCAACTTATCTTAAGAGAAGTGATCTTAGCAGAGATTGTGGAGTTCACAGGTTTTTTGGACCTTGAATTGAATCCTCGGCAATTGAAGGTATTTATTGAAGTTAAAGAAGGATGTATATTTCTGCAGGAGGAGAAATTGAAGGAGCTGATAATAATTCTTAAAAACTGTTCCTTCTCTCTTGAGGATGGTTTAGGAGGTATTTATAAAGTGATGAAGGCTAGAGGTGAACTTGGTCCTTTAATAGTATCCATTGTAAGGCCAGGCAGTTTTGATTGCGTATCAGAAAAGGCCATAGAAAGTGGGGCACCGGCAAGTCAATATAAACCCCCAAAAATCATAAGAAATCGCAAATTTGCTGATTTACTGGAAATGTCCGTTGTTGTGACTGTTTGCTTGAATTCTTTGAATGGTTAA
17	Pg_S8124.1 (PgGH3.13)	ATGCCTGAAGCTCCAAAAAATTACTCCCACAAGCTCACAGACTTAAGTGTCCTTACAGAGAAGAACAAAAAGGCTCTTCAGTTCATTGAAGATGTCACCTCTAACGCCGATGAGGTTCAGAAGCGGGTTCTTGCCGAAATTCTCTCCCGGAGTGCCCATGTAGAATACCTGCGGCGACACGGCCTCGACGGCTACACCGACAGGGACACTTTCAAAAAAATCATGCCCGTCATCAAATATGAGGATTTACAGCCTGATATCAACCGTATTGCCAATGGTGATAACTCCCCAATCCTTTGCTCTGACCCCATTTCTGAGTTCTTAACAAGCTCTGGGACATCTGGAGGAGAGAGAAAACTAATGCCCACCATTGAAGAAGAGCTAGAGAGGAGATCGCTGCTGTATAGCCTTTTGATGCCTGTGATGAACCAGTCTGTTCCTGGTCTAGACAAAGGCAAAGGAATGTATTTCTTGTTTATTAAATCTGAGGCTAAGACTCCTGGTGGCCTACTAGCTCGTCCCGTTTTAACTAGTTACTACAAAAGTTCCCATTTCAAAAACAGGCCTTATGACCCGTACACAAACTACACTAGCCCAAATGAGACCATTCTCTGCCCAGACTCCTACCAGAGCATGTACTCCCAAATGCTCTGTGGGCTATGCCTTAACAAGGAAGTCCTTAGGGTCGGAGCTGTTTTTGCCTCCGGCTTCATCCGGGCCATCCGGTTCCTTGAAAAGCACTGGTCCATTCTCTGTAACGACATCCGAACCGGAACTCTTAATCCTGAAATCATCGACCCATCGGTTAGAGAATCGGTGATGAAAATCCTCAAACCCGACACCCAATTAGCGGATTTTATTGAGGCAGAATGTAGTAAAAAGTCATGGCAAGGGATCATAACTAGGCTGTGGCCTAACACAAAGTATGTTGATGTTATTGTGACAGGGACCATGTCACAGTATATACCAACTCTTGACTACTATAGCAATGGACTCCCTCTTGTGTGCACCATGTATGCTTCTTCAGAGTGCTACTTTGGTGTCAATCTTAACCCTCTTTGCAAGCCTAGTGAAGTCTCCTATACCCTCATCCCAACCATGGCCTATTTTGAATTTTTGCCTGTCCATAGAAACAATGGTACTGTCACAAATTCCATAACCATGCCCAAATCCCTCAATGAGAAGGAACAGCAAGAATTGGTTGATCTGGCAGATGTAAAGCTCGGAGAAGAATATGAGCTTGTTGTCACCACCTATGCTGGGCTTTATAGATACAGAGTGGGAGATGTGCTTCGAGTTGCTGGATTCAAGAATAAGGCCCCCCAATTCAACTTTATTTGCCGCAAAAATGTGGTTCTAAGCATCGATTCAGACAAAACTGATGAAGTTGAACTTCAAAACGCGGTAAAAGTCGCTGTCAACCATCTGATGCCATTTGATGCTACTCTGGTTGAGTACACAAGCTATGCAGATACAACTACAATTCCGGGGCACTATGTTCTTTTCTGGGAGCTTAGTGTGAATGGATCAACCCCAATTCCTCCATCCGTTTTTGAGGATTGTTGCCTTGCAATTGAAGAGTCTCTCAACAGTGTGTACCGCCAAGGCCGTGTCTCGGACAAATCCATTGGTCCCCTTGAGATCAAGATTGTTGAATCGGGGACTTTTGATAAGCTTATGGATTATGCCATTAGCTTAGGTGCTTCAATTAACCAGTACAAGACTCCCCGGTGCGTGAAATTTGCACCAATTGTTGAGCTTCTGAACTCAAGAATTGGGTCCAACTACTTCAGTCCAAAATGCCCAAAGTGGGTTCCGGGGCATAAGCAATGGAACAACATGAACTGA
18	Pg_S1459.16 (PgGH3.11)	AATTAATATTAGTTTTCATCAATTTTAATTTATATTATGATGGACGGAAAGAAATTGGAATACAAAGGGGAGAAGGCATTGAAGGAGCTTGAGAAGCTTACGGAAAATGCCGATAAAGTTCAAGAAAATATATTGAAAGAGATCTTAAAAAGAAATGGGGAGACTGAGTATTTAAGTAAGTACATGTTGGGATCAAATGATGTTCAAGAGTTTAAAAGGTGTGTGCCAGTTATTACTTACAAGGGAATCCGTCCTTATATTCAAAGGATTGGCAATGGGGAAGACTCTGCCCTTATTACTGGCCACCCCATAACTGAGATGCTATGCAGCTCGGGGACATCTGCTGGAGAGCCGAAGCTTATGCCATCTATTGCCGAAGATCTTGATCGTCGAACCTTTCTTTATAATCTCATCATGCCAATTATCAACCAGTATATCCCAGGTCTTGATGAGGGTAAAGCCATGTTTCTCTACTTTGTCAAGGCTGAAATGTCAACCCCTTGCGGCCTACCTGCTCGGACCGTCCTAACCAGCTACTACAAGAGCAAGCACTTCAAGTTCCGGTCACGCGATCCTTACACCGATTTCACTAGCCCTGACCAAGCCATCCTTTGCAATGACAGCAACCAAAGCATGTACTGCCAATTGCTAGCCGGCCTAGTGCACCGCACCCAAGTCCTACGCCTCGGAGCCGTTTTTGCCTCAGCCTTACTCCGAGCCATATCTTTCCTTGAACGCAACTACATTAACATATGCAATGACATCCGGACCGGCCAGTTGGGGCCCAACATCACGGACCTTGAATGCCGGTCCGCCATGTCCTCCGTCCTCGTGTCGCCGGACCCACATCTTGCTGACGAGATCGAGGCCATATGTAGCCGGGCATCATGGAAGGGAATAGTGTGTCACATTTGGCCTAGGGCTAAGTACATTGAGGCAGTTGTGACAGGGTCCATGGCACAATATATACCAGCACTAGACTATTATAGTGAAGGGAAGTTGCCATTGGTTTGTACCATGTATGCATCATCTGAATGTTACTTTGGTGTGAATTTGAAGCCGTTGTATGCCCCAGCTGATGTATCTTTCACTTTATTGCCTAATATGGGTTACTTTGAGTTCATACCTTTGGGAGCGAATGGGACATTATTTTCGGACGTGGATGAAGATGAAGAGGTGCCACAAAATAAGCTAGTGGACTTGGTGGATGTTAAACTTGGTTGTTATTATGAATTGGTGGTTACTACATTTTCTGGATTATATCGATATCGAATTGGTGATGTGCTCCAAGTGAGTGGATTCCACAATCGAGCCCCGCAATTTCGGTTCATCTGCAGGCGAAACGTCCTTCTCAGCATCGACAACGACAAAACCAATGAAGAAGACCTTCACAAGAGCATCACGGTGGCCAAAAGACTCTTAGAACCCTACAATGCTCTACTTGTGGAATACAGTAGCTATGCTGACACCTCATCTGTCCCCGGTCACTATGTTATCTATTGGGAGATTATTCATTGCTCATCATCATTACTGCTAGATAATATGGTTACTAGCCAATTAGTCGATATCTCAGATGTTCTTCAAGATTGCTGCATCGCGGTGGAGGAGGAACTGGACTACAACTACAGGCGTTGTCGCGCTAATGACAAGTCCATCGGACCTCTTGAAATCCGTGTGGTGAAGCCCGGGACTTTTGAATCGTTGATGGACTTGTTTATCAACCAAGGGGGATCCATTAATCAGTATAAGACTCCAAGGTGCATCAAGCCGTCTAATGCTGCTGCACTTAAGCTGCTTAACTCTAATGTCGAGGCTTGCTTCTTTAGCCCCAGGGATCCTACGTGGAACCCTTGAGTCATGTGTTAATTAACCCGTTAATTTTGACTTGATTAGTACTCCACTTAGTAGTGAGAGTTTTAAAGACTCTCTTCTAGCTAATCAGCTTTTTAATTAAGTTTAGCTTAGATTATTATAATTAAGGTAGATTTTAAGGACTCTTATGTCATCAATGCTCATCGATCCTTTTTGTGGTAACATCTTTGGCGGAGTTTATAAGTATA
19	Pg_S7475.2 （PgGH3.2）	ATGATGGACGGAAAGAAATTGGAATACAAAGGGGAGAAGGCATTGAAGGAGCTTGAGAAGCTTACGGAAAATGCCGATGAAGTTCAAGAAAATATATTGAAAGAGATCTTAAAAAGAAATGGGGAGACTGAGTATTTAAGTAAGTACATGTTGGGATCAAATGATGTTCAAGAGTTTAAAAGGTGTGTGCCAGTTATTACTTACAAGGGCATCCGTCCTTATATTCAAAGGATTGGCAATGGGGAAGACTCTGCCCTTATTACTGGTCACCCCATAACTGAGATGCTATGCAGCTCGGGGACATCTGCTGGAGAGCCGAAGCTGATGCCATCTATTGCCGAAGATCTTGATCGTCGAACCTTTCTTTATAATCTCATCATGCCAATTATCAACCAGTATATCCCAGGTCTTGATGAGGGTAAAGCCATGTTTCTCTACTTTGTCAAGGCTGAAATGTCAACCCCTTGCGGCCTACCTGCTCGGACCGTCCTAACCAGCTACTACAAGAGCAAGCACTTCAAGTTCCGGTCACGCGATCCTTACACCGATTTCACTAGCCCTGACCAAGCCATCCTTTGCAATGACAGCAACCAAAGCATGTACTGCCAATTGCTAGCCGGCCTAGTGCACCGCACCCAAGTCCTACGCCTCGGAGCCGTTTTTGCCTCAGCCTTACTCCGAGCCATATCTTTCCTTGAACGCAACTACATTAACATATGCAATGACATCCGGACCGGCCAGTTGGGGCCCAACATCACCGACCTTGAATGCCGGTCCGCCATGTCCTCGGTCCTCGTGTCGCCGGACCCACATCTTGCTGACGAGATCGAGGCCATATGTAGCCGGGCATCATGGAAGGGAATAGTGTGTCACATTTGGCCTAGGGCTAAGTACATTGAGGCAGTTGTGACGGGGTCCATGGCACAATATATACCAGCACTAGACTATTATAGTGAAGGGAAGTTGCCATTGGTTTGTACCATGTATGCATCATCTGAATGTTACTTTGGTGTGAATTTGAAGCCTTTGTATGCCCCAGCTGATGTATCTTTCACTTTATTGCCTAATATGGGTTACTTTGAGTTCATACCTTTGGGAGCGAATGGGACATTATTTTCGGACGTGGATGAAGATGAAGAGGTGCCACAAAATAAGCTAGTGGACTTGGTGGATGTTAGACTTGGTTGTTATTATGAATTGGTGGTTACTACATTTTCTGGATTATATCGATATCGAATTGGTGATGTGCTCCAAGTGAGTGGATTCCACAATCGAGCCCCGCAATTTCGGTTCATCTGCAGGCGAAACGTCCTTCTCAGCATCGACAACGACAAAACCAATGAAGAAGACCTTCACAAGAGCATCACGGTGGCCAAAAGACTCTTAGAACCCTACAATGCTCTACTTGTGGAATACAGTAGCTATGCTGACACCTCATCTGTCCCCGGTCACTATGTTATCTATTGGGAGATTATTCATTGCTCATCATCATTGCTGCTAGATAATAACAATAATATTATGGAAAATAATAACATGGTTACTAGCCAATTAGTCGATATCTCCAATGTTCTTCAAGATTGCTGCATCGCGGTGGAGGAGGAACTGGACTATAATTACAGGCGTTGTCGCGCTAATGACAAGTCCATCGGACCTCTTGAAATCCGTGTGGTGAATCCCGGGACTTTTGAATCGTTGATGGACATGTTTATCAACCAAGGGGGATCCATTAATCAGTATAAGACTCCAAGGTGCATCAAGCCGTCTAATGCTGCTGCACTTAAGCTGCTTAACTCTAATGTCGAGGCTTGCTTCTTTAGCCCCAGGGATCCTACGTGGAACCCTTGA

20	Pg_S2144.24 (PgGH3.9)	ATGCCTGAACGCGAAAGGGAAGTTTCTGGGAAGTGTGGATCTGTCCAGTTCACCATTAATATGATGAACGAGCAGCTTGCCAAGCTGTCTAACCGGATTCTCAAACTGAATTACGG<br>CGTGGAGTACCTTAAGAAATGGTTTGGAGACATGAATGTTGAAGATATGGATGATAATGCATTAGAAATGTTGTACACCTCGTTGGTGCCTCTTGCCAGCCATGAAGATTTTGAGCC<br>TTACATTCAGAGAATTGCTAATGGGGACGCATCTCCCCTGCTCACTCAACATCCTATCACTACTCTCTCCTTAAGTTCTGGAACCACAGACGGACGACAGAAGTACGTACCCTTCAC<br>CAGCCATAGCTCCACGACTACCCTTCAGATCTATAGGTTGGCAGCAGCATATAGATCAAGGGTTTATCCACTGAAGGAAGGAGGAAGAATCCTAGAGTTCATATACAGTAGCAAAC<br>AATTGAAAACAAAAGGAGGATTAACAGCAGGGACAGCCACAACCCACTATTTCTCGAGCCAAGAGTTCAAAATAAAACAAGAAAGAACAAAAGCATTCACATGCAGCCCAGA<br>AGCAGTCATCTCAAGTGGAGACTACAAACAATCCACATATTGTCACCTCCTTCTAGGCCTCTTAGTCTCTCACGAAATAGAGTTCATAACATCCACCTTCGCTTATAGCATCATTCAA<br>GCCTTAACATTCTTCGAAGACCATTGGCGAGACCTATGTGATGACATTAAACAAGGCAGCCTCAATTCAAGGATTATCACCATACTAAAAACACAAGAATCCGTCCTAGGAATCAT<br>GTCACCAAATCCTTGTTTAGCTTCTCGGATTGAAGCAATTTGTGAAGAATTAGAAAGAGTAGGGTGGTGCGGCCTAATTCCCAAGCTATGGCCAAATGCTAAGTATGTGTACTCTAT<br>AATGACTGGCTCAATGCAACAGTATTTGAAAAAATTACAGCATTACGCTAAGGACTTGCCCTTGGTGAGTGCGGACTATGGATCTACTGAGAGTTGGATCGGAGTAAACGTGGACC<br>CTTGTGCTCCACCGGAGAATGTTAGTTTTGCAGTCATTCCCACTTTTTCGTACTTTGAGTTCATACCGCTCAATAGATACAACCATGATTGTTGCACTTCAGCTAGTACTGATGATCAT<br>GCCTACATTGAATATCAACCGATACCTCTCTCCAAACCCAAGGTTGGCCAACAATATGAGATTGTCCTAACTACTTTTACAGGGCTTTATAGGTACATATTAGGAGATGTGGTGGAA<br>GTGACTGGTTTTTACAAAGAGACTCCCAAATTGAACTTCATATGCAGGAGAAAGCTAATTTTGACCATAAATATCGACAAAAATACCGAAAAAGACCTTCAACTGGTGGTAGACA<br>AGGGTTCACAGTTGCTGAGCAGGATGGCAAGAGCGGAGGTGGTTGATTTCACTAGCCATGCTGACGTAGAAAACCAACCGGGGCAGTACATAATTTACTGGGAGATCAAAGGAG<br>ACGTCGAAGAAGACGTTCTAAGAGAGTGTTGCCAAGTAATGGACGCATCATTTGTGGATCATGGATATGTTGTGTCCAGAAGAACCAATTCGATAGGACCGCTAGAGCTTTGCATT<br>TTGGAAAGGGGCACTTTTAAGAAGATTTTGGAGCATTTCTTAGGAAATGGAGCAGCATTGAGCCAGTTCAAGACCCCAAGGTGCACTAGCAACCAAGTGTTGCTAACAATTCTCA<br>ATTCATGCATCATTAAAAGGTTTCACAGTACTGCGTACGGGTGA
21	Pg_S5926.3 (PgGH3.14)	ATGACGAGCAATGAAAATAGGAGCAGCAGTAGCGTCGAGATGGACGATGACGTTGTGAGTTGGTTCGAAGACGTAGCTGAGAGCGCAGGGCTTGTCCAGACGCAGACGCTTAG<br>CCGGATTCTCAAACTGAATTACGGCGTGGAGTACCTTAAGAAATGGTTTGGAGACATGAATGTTGAAGATATGGATGATAATGCATTAGAAATGTTGTACACCTCGTTGGTGCCTCT<br>TGCCAGCCATGCAGATTTGGAGCCTTACATTCAGAGAATTGCTAATGGGGACGCATCTCCCCTGCTCACACAACATCCTATCACTACTCTCTCCTTAAGTTCTGGAACCACAGACGG<br>ACGACAGAAGTACGTACCCTTCACTAGCCATAGCTCCACGACTACCCTTCAGATTTATAGGGTGGCAGCAGCATATAGATCAAGGGTTTATCCACTGAAGGAAGGAGGAAGAATC<br>CTAGAGTTCATATACAGTAGCAAACAATTGAAAACAAAAGGAGGATTAACAGCAGGGACAGCCACAACCCACTATTTCTCGAGCCAAGAGTTCAAAATAAAACAAGAAAGAAC<br>AAAAGCATTCACATGCAGCCCAGAAGCAGTCATCTCAAGTGGAGACTACAAACAATCCACATATTGTCACCTCCTTCTAGGCCTCTTAGTCTCTCACGAAATAGAGTTCATAACAT<br>CCACCTTCGCTTATAGCATCATTCAAGCCTTAACATTCTTCGAAGACCATTGGCGAGACCTATGTGATGACATTAAACAAGGCAGCCTCAATTCAAGGATTATCACCATACAAAAA<br>ACACGAGAATCCGTCCTAGGAATCATGTCACCAAATCCTTGTTTAGCTTCTAGGATTGAAGCTATTTGTGAAGAATTAGAAAGAGTAGGGTGGTGCGGCCTAATTCCCAAGCTATG<br>GCCAAATGCTAAGTATGTGTACTCTATAATGACTGGCTCAATGCAACAGTATTTGAAAAAATTACGACATTACGCTAAGGACTTGCCCTTGGTGAGTGCGGACTATGGATCTACTGA<br>GAGTTGGATCGGAGTAAACGTGGACCCTTGTGCTCCACCGGAGAATGTTAGTTTTGCAGTCATTCCCACTTTTTCGTACTTTGAGTTCATACCACTCAATAGATACAATCATGATTGT<br>TGCACTTCAGCTAGTACTGATGATCATGCCTACATTGAAGATCAACCGATACCTCTCTCCAAACTCAAGGTTGGCCAACAATATGAGATTGTCCTAACTACTTTTACAGGCAAGTAA

Supplementary Table S2. Orthologous proteins from other species for phylogenetic tree construction

**Gene**	**Species**	**Accession No**
AtJAR1	*Arabidopsis thaliana*	BAH19469.1
AtGH3.12	*A. thaliana*	Q9LYU4.1
OsJAR1	*Oryza sativa*	Q6I581.1
OsJAR2	*O. sativa*	Q5NAZ7.2
PhJAR1	*Panicum hallii*	XP_025807560.1
HuJAR1	*Herrania umbratical*	XP_021280203.1
QsJAR1	*Quercus suber*	XP_023873074.1
SlJAR1	*Solanum lycopersicum*	XP_004248075.1
AcJAR1	*Actinidia chinensis*	PSS24204.1
CsJAR4	*Camellia sinensis*	XP_028096856.1
DcJAR1	*Daucus carota*	XP_017244523.1
NaJAR1	*Nicotiana attenuata*	XP_019263840.1
NnJAR1	*Nelumbo nucifera*	XP_010278303.1
NtJAR1	*Nicotiana tabacum*	XP_016501463.1
NtJAR6	*N. tomentosiformis*	XP_009601287.1
TcJAR1	*Theobroma cacao*	XP_017971785.1
OeJAR1	*Olea europaea*	CAA3002169.1
CaJAR1	*Capsicum annuum*	XP_016545524.1
VvJAR1	*Vitis vinifera*	XP_002272560.1
PmJAR1	*Prunus mume*	XP_008230245.1
PpJAR1	*Prunus persica*	XP_007217151.1
PdJAR4	*Prunus dulcis*	XP_034204082.1
StJAR1	*Senna tora*	KAF7826151.1
PaJAR1	*Prunus avium*	XP_021807979.1
AiJAR1	*Arachis ipaensis*	XP_016205284.1
ItJAR4	*Ipomoea triloba*	XP_031127272.1
QlJAR4	*Quercus lobata*	XP_030965499.1
SmJAR1	*Selaginella moellendorffii*	XP_002976207.1

Supplementary Table S3. Effect of JA-Ile concentration on the growth of ginseng hairy roots

JA-Ile Concentration μmol L^{-1}	Hairy Roots Growth Parameters			
	Average Root Length (mm)	Fresh Weight (g)	Dry Weight (g)	Growth Ratio
0	4.12 ± 0.15 [a]	9.61 ± 0.22 [a]	0.91 ± 0.25 [a]	8.33 ± 0.28 [a]
1	4.23 ± 0.21 [a]	9.73 ± 0.31 [a]	0.96 ± 0.11 [a]	8.36 ± 0.37 [a]
10	4.05 ± 0.17 [a]	8.91 ± 0.45 [b]	0.87 ± 0.15 [b]	7.74 ± 0.46 [a]
20	3.71 ± 0.18 [b]	8.22 ± 0.57 [c]	0.76 ± 0.21 [b]	7.39 ± 0.51 [b]

After the preculture and JA-Ile treatment, the morphology and characteristics of ginseng hairy roots including length were monitored. The fresh weight (FW) and dry weight (DW) of each sample were measured. The growth ratio (GR, in percentage) of each culture was obtained by dividing the difference between the final FW and initial FW by the initial FW. Data are average values of three replicates ± standard deviation (SD). Means in each column with the same letters are not significantly ($p < 0.05$) different based on Duncan's Multiple Range Test.

Supplementary Table S4. Cloning and expressing primers

Gene	Primer	Sequence (5′→3′)
PgJAR1	Forward	ATGTTGGAAACTATGGAGAA
	Reverse	TCAATCAAAAGCAGTACTAAAG
PgJAR1-E	Forward	GGATCCATGTTGGAAACTATGGAGAA
	Reverse	TCAATCAAAAGCAGTACTAAAG
PgJAR1-TE	Forward	GGACTCTTGACCATGTTGGAAACTATGGAGAA
	Reverse	TCGCCTTTGGAAGTTGAATGCCTCAATCAAAAGCAGTACTAAAG
ProPgJAR1	Forward	ATTTTTACAAATAAAAATAATT
	Reverse	CTCCATAGTTTCCAACATTGAA

Note: PgJAR1, primers for gene cloning; PgJAR1-E, primers for prokaryotic expression vector construction (pET23a-PgJAR1); PgJAR1-TE, primers for transient expression vector construction (pCAMBIA1302-PgJAR1); *ProPgJAR1*, primers for promoter of *PgJAR1*.

Supplementary Table S5. Primers of the selected genes verified by qRT-PCR analysis.

Gene	Primer	Sequence (5′→3′)
PgOPR	Forward	GATGGCTCTAGCGTTGCAGA
	Reverse	TGACTGTTAACACCTACCGGC
PgJAR1	Forward	GCGGACCTACCGCTATTGAG
	Reverse	ACAGCAAAAGTTGCCGACTC
PgDDS	Forward	CCCTGCAGTGCCTACTGTTA
	Reverse	CTCCCAAACTGCGAAACCAC
PgCYP716A47	Forward	TCCACATGACACGCACAAGA
	Reverse	ATTCCCGGACACATTCGAGG
PgUGT74AE2	Forward	CTCCTGACATCAAACTCGTC
	Reverse	TGCTTCGAGTTATGGGTACA
PgCYP716A53v2	Forward	TGCCTCGGGGAACAAATTCA
	Reverse	TCCGCAAGGCTTTGCTTTTC
β-actin	Forward	TGCCCCAGAAGAGCACCCTGT
	Reverse	AGCATACAGGGAAAGATCGGCTTGA
